# Chemerin is secreted by the chicken oviduct, accumulates in egg albumen and could promote embryo development

**DOI:** 10.1038/s41598-022-12961-4

**Published:** 2022-05-29

**Authors:** Anthony Estienne, Adeline Brossaud, Christelle Ramé, Ophélie Bernardi, Maxime Reverchon, Christophe Rat, Joël Delaveau, Emilie Chambellon, Emmanuelle Helloin, Pascal Froment, Joëlle Dupont

**Affiliations:** 1 Centre National de la Recherche Scientifique, Institut Français du Cheval et de l’Equitation, Institut National de Recherche pour l’Agriculture, l’Alimentation et l’Environnement, INRAE, Université de Tours, Physiologie de la Reproduction et des Comportements, UMR85, 37380 Nouzilly, France; 2 SYSAAF-Syndicat des Sélectionneurs Avicoles et Aquacoles Français, Centre INRA Val de Loire, 37380 Nouzilly, France; 3 Institut National de Recherche pour l’Agriculture, l’Alimentation et l’Environnement-Unité Expérimentale du Pôle d’Expérimentation Avicole de Tours UEPEAT 1295, 37380 Nouzilly, France; 4 Institut National de Recherche Pour l’Agriculture, l’Alimentation et l’Environnement-Unité Infectiologie et Santé Publique, Université de Tours, Nouzilly, France

**Keywords:** Biotechnology, Physiology

## Abstract

Understanding of the distribution of chemerin and its receptors, Chemokine-like Receptor 1 (CMKLR1), G Protein-coupled Receptor 1 (GPR1) and Chemokine (C–C motif) receptor-like 2 (CCRL2), in the egg and the embryonic annexes is currently lacking, and their role during embryogenesis remains unknown. By immunoblot using monoclonal anti-chicken antibodies and Enzyme Linked Immunosorbent Assays (ELISA), we found that chemerin is expressed 10 times higher in albumen eggs than in blood plasma, and it is also abundant in the perivitelline membrane but undetectable in yolk. Chicken chemerin can inhibit bacterial growth. By Reverse Transcription—quantitative Polymerisation Chain Reaction (RT-qPCR), western-blot, and immunofluorescence, we show that chemerin is locally produced by the oviduct magnum that participates in albumen formation. Using cultures of magnum explants, we demonstrate that progesterone (P4) and oestradiol (E2) treatment increases chemerin secretion into cultured media and expression in magnum. Chemerin and its three receptors are present in amniotic and Chorio Allantoic Membranes (CAM). Only CMKLR1 expression decreased from embryonic day (ED) 7 to ED11 and remained low until ED18. Chemerin concentrations strongly increased in amniotic fluid at D14 when egg albumen crossed the amniotic membrane. In ovo injections of neutralising chemerin and CMKLR1 antibodies (0.01, 0.1 and 1 µg) increased embryo mortality, which occurred mainly at ED12-13, in a dose-dependent manner. Chemerin treatment increased primary CAM viability. Finally, chemerin and CMKLR1 inhibition within the CAM led to a decrease in blood vessel development and associated angiogenic gene expression. Our results show an important function of the chemerin system during embryo development in chickens, suggesting the potential use of this adipokine as a predictive marker for egg fertility or hatchability.

## Introduction

Chemerin is a hormone belonging to the family of adipokines and hepatokines, a class of molecules playing an important role in numerous physiological processes such as homeostasis, inflammation, immunity, angiogenesis, metabolism and reproduction^1^. It is also known as Tazarotene-Induced Gene 2 (TIG2) and Retinoic Acid Receptor RESponder protein 2 (RARRES2) which was first discovered in 1997 as a novel retinoid-responsive gene in skin^2^. Later, it was identified in human inflammatory fluids as a ligand of Chemerin Receptor 23 (ChemR23)^3^, a G protein-coupled receptor formerly discovered in 1996 under the name of chemokine-like receptor 1 (CMKLR1) for its high sequence and structural homology to the seven transmembrane G-protein-linked chemokine receptors^4^. Chemerin is first secreted in the form of an 18 kDa-inactive preprochemerin of 163 amino acids, with the secreted form being 20 amino acids shorter due to the cleavage of the C-terminus by inflammatory and coagulation serine proteases^1,5^. Chemerin can bind three G-coupled receptors with seven transmembrane domains, CMKLR1^1^, G Protein Receptor 1 (GPR1)^1^ and C–C chemokine receptor-like 2 (CCRL2)^6^.

In mammals, chemerin has been extensively studied in the female reproductive function in many models^7–9^. In human and bovine ovaries, chemerin inhibits Insulin-like Growth Factor 1 (IGF-1)-induced steroidogenesis in primary granulosa cells^10,11^. Similar results were obtained in the 5α-dihydrotestosterone (DHT)-induced rat model that mimics the reproductive and metabolic phenotypes of human PolyCystic Ovarian Syndrome (PCOS). In this latter model, chemerin suppressed FSH-induced progesterone (P4) and estradiol (E2) secretion into cultured pre-antral follicles and granulosa cells, suggesting that chemerin is a negative regulator of Follicle Stimulating Hormnone (FSH) -induced follicular steroidogenesis and could contribute to the pathogenesis of PCOS^12^. Moreover, in mice Knock-Out for *CMKLR1* gene, the effects of chronic DHT treatment on ovarian function in experimental PCOS inducing are largely reduced, suggesting a role of the chemerin system in PCOS pathology^13^. This hypothesis has also been made for humans^14,15^. In mammals, chemerin and its receptors are expressed in other parts of the reproductive tract including the uterus^16–18^, where they could be involved in vascular remodelling during early human pregnancy^19^.

In birds, chemerin and its receptors are also expressed within hen ovaries ^20,21^ and a significant positive correlation between chemerin expressional levels in granulosa cells and the weight of the F1 preovulatory follicle exists^22^. In turkeys, the chemerin system is also present in the theca and granulosa cells of ovarian follicles, with higher expression levels in theca cells^20^. Moreover, results have shown that chemerin plasma concentrations decreased during the laying period in the latter model^20^. In chickens, our team demonstrated that plasma chemerin concentrations were negatively associated with egg hatchability, suggesting a potential role of this adipokine on egg and/or embryo development^22^. After in vivo experiments on fresh rooster sperm incubated with recombinant chicken chemerin and used for artificial insemination (AI), we observed a negative effect of chemerin on egg fertility for the first 3 days after AI, suggesting an impact of this hormone on early embryo development (EED)^23^. In addition, in turkeys, we found that chemerin expression in granulosa cells increases in the mature follicle immediately before ovulation, thus, potentially influencing EED^20^. The latter results led us to investigate chemerin expression and its role in egg and embryo development in chickens as well as origin of production within the oviduct of the hen.

Thus, the objectives of the present study were to (1) determine the expression of chemerin and its receptors in the oviduct and the different compartments of eggs (perivitelline membranes, yolk, and albumen) and (2) investigate the role of chemerin in embryo development by using specific tools including chicken recombinant chemerin and antibodies against chicken chemerin and its receptors.

## Materials and methods

### Ethial statement

All experiments were approved by the Ethics Committee in Animal Experimentation of Val de Loire (CEEA Vdl) (certificate of authorisation to experiment on living animals APAFIS number 10237-201706151202940v3). The CEEA vdl is registered by the National Committee ‘Comité National de Réflexion Ethique sur l'Expérimentation Animale’ under the number 19. All experiments were performed in accordance with the European Communities Council Directive 2010/63/UE. The study is reported in accordance with ARRIVE guidelines.

### Animals and samples collection

**Figure 1:**
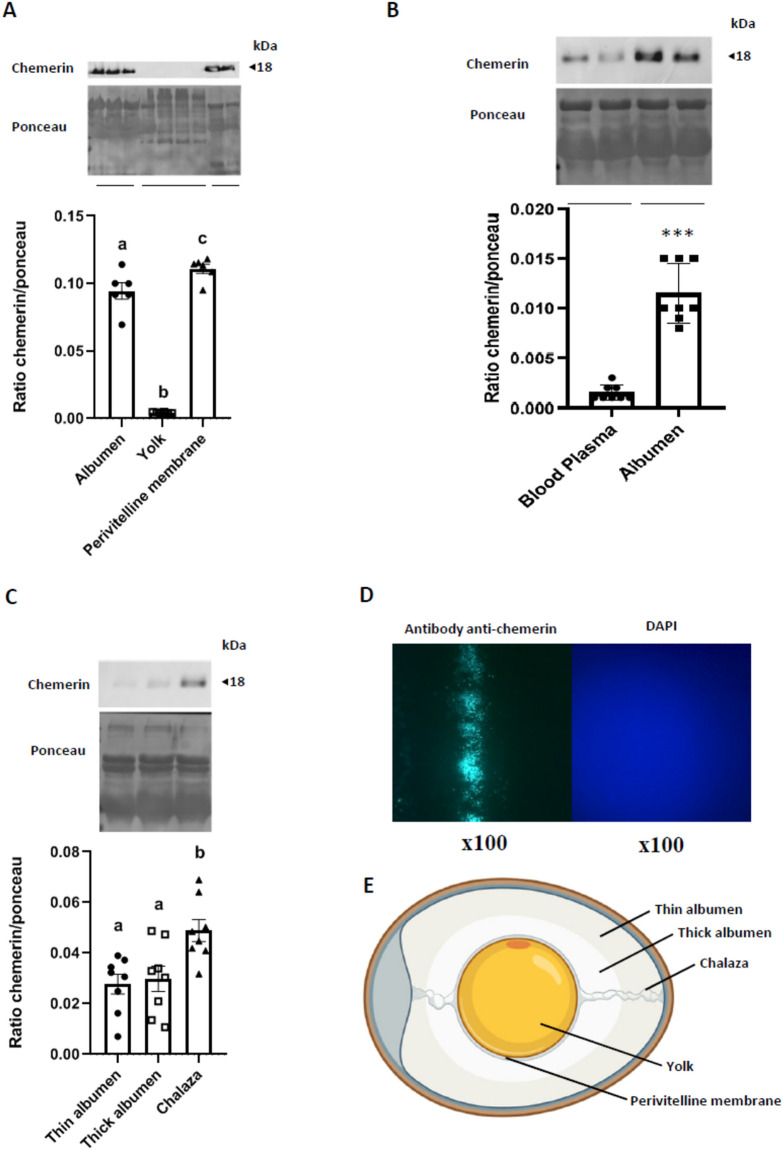
Chemerin accumulation within the egg. Ten hens were randomly selected to collect blood plasma, and six eggs were randomly selected to sample thick and thin albumen, perivitelline membrane, yolk, and chalazas. (**A**) Protein abundance of chemerin detected by western blotting within the albumen (mix of thin and thick), yolk, and the perivitelline membrane in the eggs (n = 6). (**B**) Protein abundance of chemerin detected by western blotting within the blood plasma and the albumen (mix of thin and thick) of hens (n = 8 animals and eggs). (**C**) Protein abundance of chemerin detected by western blotting within the thin and thick albumen, and the chalaza of eggs (n = 10). (**D**) Immunofluorescence for chemerin qualitative detection within the perivitelline membrane (magnification ×100). (**E**) Localisation of different components (thin and thick albumen, yolk, chalaza, and perivitelline membrane. The values are expressed as mean ± standard errors of means. Different letters indicate significant differences at p < 0.05 and ***indicate significant differences at p < 0.001.

One hundred broiler breeder unfertilised eggs (Cobb 500, HendrixGenetics, Saint Laurent de la Plaine, France) were used to collect thin and thick egg white (albumen), chalaza, yolk, and perivitelline membrane (Fig. 1E). The chicken egg white consists of two layers—thin and thick albumen (Fig. 1E) with the thin albumen surrounding the thick albumen. Thick albumen immediately surrounds the yolk, acting as a cushion for the yolk. The chalaza is found in the thick egg white attached to the end of the yolk (Fig. 1E). The avian perivitelline membrane, which is an investment homologous to the mammalian zona pellucida, is found between the surface of the oocyte and the apical surface of ovarian granulosa cells (Fig. 1E).

**Figure 2:**
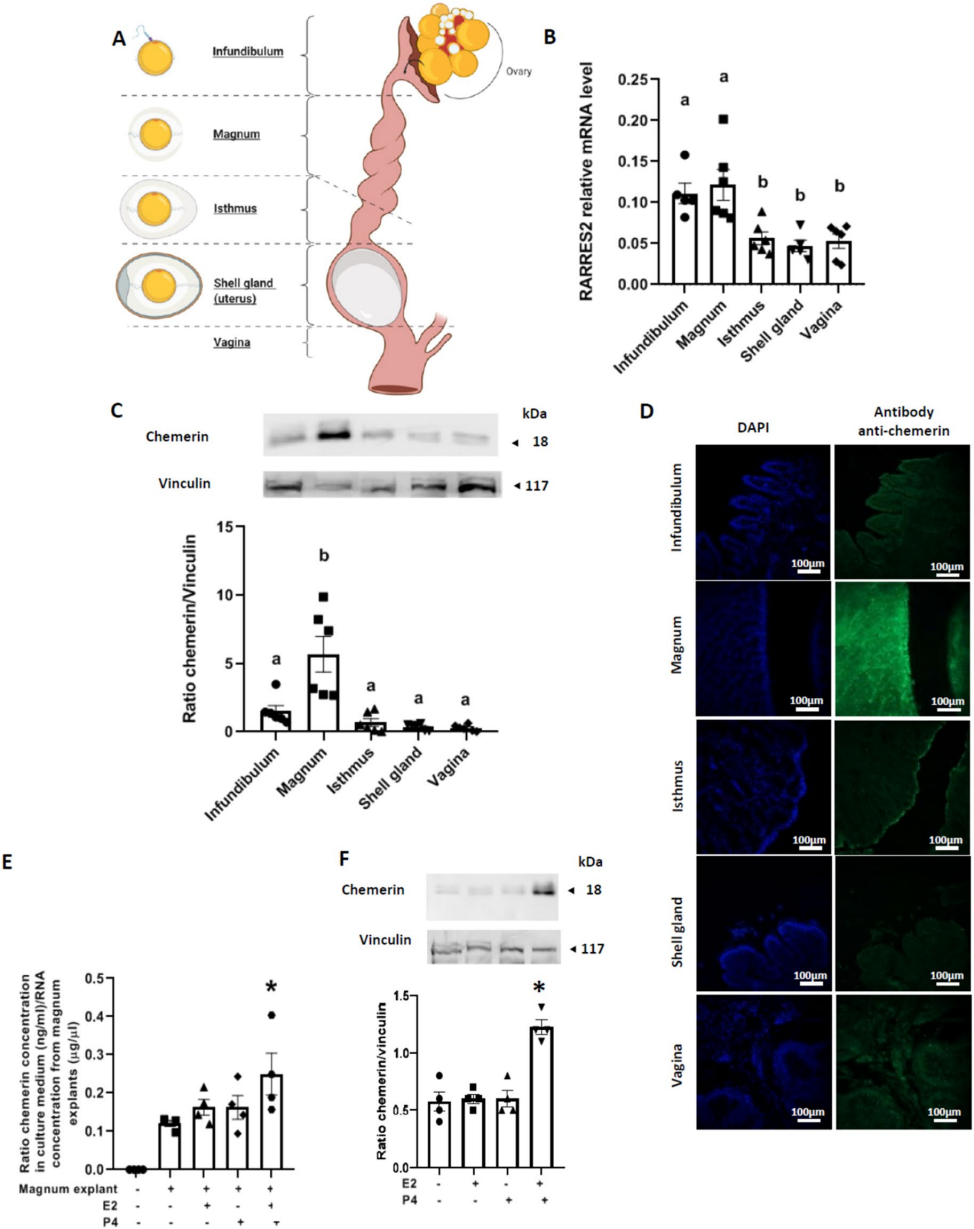
Origin of chemerin production in albumen. A total of 10 hens were randomly selected during the laying period and euthanised to collect samples of their oviducts. (**A**) Localisation of different parts of the avian oviduct. (**B**) RARRES2 relative expression in the different parts of the oviduct of hens (infundibulum, magnum, isthmus, shell gland and vagina) quantified by RT-qPCR (n = 6). (**C**) Protein abundance of chemerin detected by western blotting within the infundibulum, magnum, isthmus, shell gland and vagina of hens (n = 6). (**D**) Immunofluorescence for chemerin qualitative detection within the hen’s oviduct (scale 100 μm). (**E**) Ratio between chemerin concentration secreted in the culture medium by magnum explants (measured by Enzyme Linked Immunosorbent Assay) and the mRNA quantity extracted from the same explants under basal conditions or stimulated for 48 h with oestradiol (E2) (10^–8^ M) and/or progesterone (P4) (10^–8^ M) (n = 4). (**F**) Values are expressed as mean ± standard errors of means. Different letters indicate significant differences at p < 0.05, and *indicates significant differences compared to the condition magnum explant without hormonal stimulation at p < 0.05.

Ten female broiler breeder chicks (Cobb 500 breed) from Hendrix Genetics (Saint Laurent de la Plaine, France) were kept in a pen of 3 m^2^ with thermostatically controlled air inlets and a dynamic cross-ventilation system. The pen was equipped with a hanging feeder, drip nipples, and 5 kg of fresh wood shavings as litter, and hens were fed ad libitum. Animals were reared at Pôle Expérimental Avicole de Tours (PEAT, INRAe, Nouzilly, France) under conventional breeding conditions (Cobb-Vantress, 2008): 24 h of light on arrival, with day length being reduced to approximately 8 h at 2 days of age, then kept constant until the age of photo-stimulation (21st week). From 21 weeks of age, there was a gradual increase in exposure to light up to 15 h per day at 25 weeks to induce laying. The animals were maintained under this light regime until the end of the experiment. At 36 weeks, six hens were selected randomly, euthanised by electrical stunning and bled out for blood sample collection, as recommended by the ethical committee. Their oviducts were collected, and a sample of each part (infundibulum, magnum, isthmus, shell gland (uterus), and vagina) (Fig. 2A) was collected and fixed with formalin for 48 h or stored at − 80 °C until use.

Fifty broiler breeder fertile chicken eggs (Cobb 500, Hendrix Genetics, Saint Laurent de la Plaine, France) were conventionally incubated at the PEAT experimental unit (INRAe, Centre Val de Loire, Nouzilly, France) according to the following protocol. Eggs were stored in a room at 15–16 °C and 80–85% humidity for 1 week. Then, all eggs were placed in alternative rows on each shelf of the incubator. They were maintained at 37.8 °C and 56% relative humidity and automatically turned every hour. At days 7 and 14 of incubation, all eggs were candled, and infertile eggs, along with dead embryo eggs, were eliminated at day 18 of incubation. Eight fertile eggs were retrieved at 7 (ED7), 9 (ED9), 11 (ED11), 14 (ED14), and 18 (ED18) days of incubation. Embryos from each stage (ED7, ED9, ED11, ED14, and ED18) were taken and sacrificed by decapitation, and the annexes were immediately sampled (amniotic membrane, amniotic fluid, chorioallantoic membrane) (Fig. 4B), snap-frozen and stored at − 80 °C until use.

### Recombinant chicken chemerin and chicken chemerin receptors antibodies

The recombinant chicken chemerin protein (full length, rRARRES2) was obtained from the *Gallus gallus* sequence (NM_001277476.1), produced in *Escherichia coli* and purified by a chromatography column-based on His-Tag under denaturation condition (Agro-Bio, La Ferté Saint Aubin, France). Monoclonal chicken chemerin antibodies were produced by AgroBio (La Ferté Saint Aubin, France), and their specificity was tested as previously described^24^. Specific antibodies against chicken CMKLR1 were produced by AgroBio (Orleans, France). Briefly, two peptides corresponding to 20 amino-terminal residues (DDSDTYDYLDYTYEEPGSV, CMKLR1-20) and 18 carboxy-terminal residues (HRSFSKMSSMTEKETTVL, CMKLR1-18) of chicken CMKLR1 were conjugated to keyhole limpet hemocyanin using sulfhydryl chemistry (Sigma Genosys, Woodlands, TX). One hundred fifty micrograms of both conjugated CMKLR1-20 and CMKLR1-18 were emulsified with an equal volume of complete Freund's adjuvant and injected into two New Zealand White rabbits. Secondary immunisations were performed at days 14, 34, 57, 77, and 98 (relative to primary immunisation) followed by bleeding on day 105. Blood samples were collected from an ear vein for titre testing in microtiter plates at days 42, 63, and 84. The specific humoral immune response to targeted antigens (CMKLR1-20 and CMKLR1-18) was evaluated by a direct ELISA using 96-well microtiter plates. The Immunoglobulins G (IgG) from the antiserums were purified by protein G-affinity chromatography with the use of a HiTrap protein-G column (GE Healthcare), and the protein content was quantified with the Bicinchoninic Acid (BCA) kit (Sigma-Aldrich). The specificity and activity of chicken CMKLR1 antibodies have previously been described^23^. In the same manner, specific antibodies against chicken GPR1 were produced by AgroBio (Orleans, France). Two peptides corresponding to 18 amino-terminal residues (CYSYFYDLPEEEESPQST) and 18 carboxy-terminal residues (VELSAHHDENLHDLLQDC) of chicken GPR1 were conjugated to keyhole limpet hemocyanin, using sulfhydryl chemistry (Sigma Genosys, Woodlands, TX). All antibodies were used at 1/1000 dilution in western blotting.

### In vitro culture of magnum explants

Our in-vitro culture model of magnum explant has been adapted from Kasperczyk et al*.*^25^. Briefly, magnums from eight laying hens were collected by dissection after slaughtering and kept in saline solution for about 30 min at room temperature before use. For each culture, one piece of magnum tissue was washed twice in Phosphate Buffered Saline (PBS) plus penicillin (100 U/ml)/streptomycin (100 μg/ml) and subsequently finely cut in small pieces of 1 mm of diameter. Between two and three explants were cultured in each well of a 24-well culture plate and incubated with E2 and/or P4 at 37 °C in Gold Keratinocyte growth Basal Medium (KBMTM, Lonza, ref 00192151), supplemented with SingleQuotsTM Supplements and growth factors (Lonza, ref 00192152) without serum. To determine the amount of secreted chemerin within the culture medium, explants were subjected to 48 h of stimulation. At the end of the experiment, the conditioned culture medium was kept at – 20 °C before use for chemerin assays. Eight independent cultures were performed.

### In-ovo injection experiments

A total of 500 broilers eggs were injected at embryonic day 7 (ED7) of incubation. The eggs were randomly assigned to eight treatment groups: control (PBS), IgG, chemerin antibody at 0.01, 0.1 and 1 μg/mL and CMKLR1 antibody at 0.01, 0.1 and 1 μg/mL. The eggshell was disinfected by spraying 1.5% hydrogen peroxide and punctured using a sterile needle. One hundred microliters of either PBS or treatment were injected into the albumen and the injected eggs were incubated under standard hatchery settings. Daily, we assessed the level of embryonic mortality by candling and then breaking eggs when the embryo appeared dead from the 7th day of incubation until the 14th day of incubation, when the experiment was stopped.

### In-vitro culture of amniotic and allantoic cells

Twelve broiler eggs were sampled at ED 7 and 14 of incubation in order to recover amniotic and allantoic membranes by dissection. After collection, tissues were washed three times in PBS and then digested with collagenase A 0.3% (Roche Diagnostic, Mannheim, Germany) for 30 min in a warm bath at 37 °C. To remove most of the red blood cells, the pellet was centrifuged (400*g*, 20 min) on a two-layer discontinuous Percoll gradient (40%, 60% in Ham medium, GibcoBRL; Life Technologies, Cergy Pontoise, France). The 40% fraction was collected and washed with fresh Dulbecco's Modified Eagle Medium (DMEM), cells were counted in a haemocytometer, and cell viability was determined using Trypan Blue dye exclusion (Sigma). Cells were cultured in DMEM medium supplemented with 20 mmol/L Hepes, penicillin (100 U/ml), streptomycin (100 mg/L), and L-glutamine (3 mmol/L). The cells were cultured for 48 h with or without chicken recombinant chemerin at 1, 25, 2.5, 5, 25, 50 μg/mL and with or without IGF1 at 10^–9^ M. All cultures were kept under a water-saturated atmosphere of 95% air, 5% CO_2_ at 37 °C.

### Cell counting kit-8 (CCK8) assay

Cell viability was evaluated using the Cell Counting Kit-8 (CCK8, Sigma Aldrich, Saint Quentin-Fallavier, France). Cells were previously dispensed into sterile 96-well cell culture plates to obtain about 4000 cells per well. Twenty-four hours later, cells adhering to the plate were stimulated with increasing concentrations of chemerin (0, 1.25, 2.5, 5, 25 or 50 μg/mL) with or without IGF1 (10^–9^ M) for at least 24 h. The supernatant was then removed and replaced with DMEM medium containing 100 μL of CCK8 10%. After 3.5 h of incubation at 37 °C in a water-saturated atmosphere with 5% CO_2_, the absorbance at 450 nm was measured using a microplate reader. The optic densities obtained were then related to a standard range previously defined.

### Chemerin enzyme linked immunosorbent assay (ELISA assay)

Chemerin concentrations were measured by Enzyme Linked Immunosorbent Assays (ELISA) using commercial kits [chicken chemerin: MBS738819 (sensitivity 0.1 ng/mL, MyBioSource, San Diego, USA)] according to the manufacturer’s instructions. All assays were performed in 96-well plates and absorbance was measured at 450 nm using a Microplate Reader (Tecan, Magellan, Männedorf, Switzerland). A standard curve was drawn for the determination of hormone levels. The experiment was performed following the manufacturer’s protocol with an intra-assay coefficient variation of < 15%.

### Reverse transcription and quantitative PCR (RT-qPCR)

Total RNA was extracted from the infundibulum, magnum, isthmus, shell gland and vagina of 36 week-old hens (six animals) by the homogenisation of 100 mg of tissue in lysis buffer reagent, using a total RNA extraction kit according to the manufacturer’s recommendations (NucleoSpin RNA, Macherey–Nagel, Hoerdt, France). The complementary DNA (cDNA) was generated by reverse transcription (RT) of total RNA (1 µg) in a mixture comprised of 0.5 mM of each deoxyribonucleotide triphosphate (dATP, dGTP, dCTP and dTTP), 2 M of RT buffer, 15 µg/µL of oligodT, 0.125 U of ribonuclease inhibitor, and 0.05 U of Moloney murine leukaemia virus reverse transcriptase (MMLV) for 1 h at 37 °C. Real-time PCR was performed using the MyiQ Cycle device (Bio-Rad, Marnes-la-Coquette, France) in a mixture containing SYBR Green Supermix 1× reagent (Bio-Rad, Marnes la Coquette, France), 250 nM specific chicken primers (Invitrogen by Life Technologies, Villebon sur Yvette, France, see Table 1) and 5 µL of cDNA (diluted fivefold) for a total volume of 20 µL.

**Table 1:** List of primers used for qRT-PCR.

Gene	Primer forward	Primer reverse	Efficiency
*RARRES2*	CGCGTGGTGAAGGATGTG	CGACTGCTCCCTAAAGAGGAACT	1.90
*CMKLR1*	CGGTCAACGCCATTTGGT	GGGTAGGAAGATGTTGAAGGAA	1.95
*GPR1*	ACCTGCCTGAGGAAGAAGAA	AAAGGCCAGTGGAAGCCCAT	2.00
*CCRL2*	CACGCAGTGTTTGCTTTAAAAGC	CAACAGCCCACGTGACAATG	1.92
*CALD1*	TTGAGCGTCGCAGAGAACTT	AGGCGTTTTTGGCGTCTTTC	2.04
*TEM8*	TGAGAGGGAGGCCAATCGGTCA	GCAGCGGCCCTTGTCTCCTG	2.00
*EPHRIN B2*	TTCCCCACAACACACCACAA	TCGCCGCTGACCTTTTCATA	1.95
*CXCR4*	TGTGTATGTGGGTGTCTGGC	CAGCCAGTTGTCATGAGGGT	1.94
*TIE2*	CTGAGTGCTGGGATGCTACC	GTGATAGACTCACGGGAGCG	2.00
*GAPDH*	TGCTGCCCAGAACATCATCC	ATCAGCAGCAGCCTTCACTACC	1.98
*EEF1a*	AGCAGACTTTGTGACCTTGCC	TGACATGAGACAGACGGTTGC	1.95
*Β Actin*	ACGGAACCACAGTTTATCATC	GTCCCAGTCTTCAACTATACC	2.02

The samples were duplicated on the same plate, and the following PCR procedure was used. After an incubation of 2 min at 50 °C and a denaturation step of 10 min at 95 °C, samples were subjected to 40 cycles (30 s at 95 °C, 30 s at 60 °C and 30 s at 72 °C). The expression levels of messenger RNA were standardised to three reference genes (*Eukaryotic translation elongation factor 1 alpha 1 (EEF1), Glyceraldehyde-3-Phosphate Dehydrogenase (GAPDH)* and *β Actin*). Chemerin expression was calculated according to primer efficiency (E) and quantification cycle (Cq), where expression = E^− Cq^. Then, the relative expression of the target gene to the geometric mean of the three reference genes was analysed.

### Western blot

Samples collected from non-incubated eggs, oviducts and annexes from incubated eggs at ED7 to ED18 were lysed using an Ultraturax (Invitrogen™ by Life Technologies™, Villebon sur Yvette, France) in lysis buffer (Tris 1 M (pH 7.4), NaCl 0.15 M, EDTA 1.3 mM, EGTA 1 mM, VO 43–23 mM, NaF 0.1 M, NH_2_PO_4_ 1%, Triton 0.5%). The lysates were centrifuged for 20 min at 17,000*g* at 4 °C, and the supernatant containing proteins was collected and kept on ice. The protein concentration of lysates was measured using the BCA protein assay (Interchim, Montluçon, France). Tissue proteic lysates (60 µg) were mixed with Laemmli buffer 5× and proteins were denatured for 5 min by heat shock at 95 °C. Subsequently, proteins were loaded in an electrophoresis sodium dodecyl sulphate–polyacrylamide gel [12% for high protein weight (110–20 kDa) and 15% for low protein weight (< 20 kDa)] and then transferred to a nitrocellulose membrane. Membranes were blocked with Tris-Buffered Saline Tween buffer containing 0.05% Tween 20 and 5% milk for 30 min at room temperature. Membranes were incubated overnight at 4 °C with the appropriate primary antibody (Cf above for anti-chemerin, anti-CMKLR1, and anti-GPR1 antibodies; anti-vinculin antibody^26^; anti-CCRL2 antibody^27^). Then, membranes were incubated for 90 min at room temperature with a Horse Radish Peroxidase-conjugated anti-rabbit or anti-mouse IgG. Proteins of interest were detected by enhanced chemiluminescence (ECL, Western Lightning Plus-ECL, Perkin Elmer, Villebon-sur-Yvette, France) with a G-box SynGene (Ozyme, St Quentin en Yvelines, France) and the GeneSnap software (Ozyme, St Quentin en Yvelines, France) and subsequently quantified with the GeneTools software. The results were expressed as the intensity signal in arbitrary units after normalisation of target proteins signals with housekeeping protein signals (vinculin) or the total protein amount (Ponceau).

### Immunofluorescence

For chemerin immunodetection experiments within the oviduct, samples of the infundibulum, the magnum, the isthmus, the shell gland, and the vagina from adult hens (36 week) were fixed in formalin, paraffin-embedded and finally sectioned (7 µm) as described in Guibert et al.^28^. Sections were dewaxed and rehydrated in xylene in decreasing concentrations of alcohol (100%, 90%, and 75%). Antigen retrieval was performed by steaming the sections in a microwave in citrate buffer (0.01 M) with a pH of 6.0 for 5 min, then cooling for 20 min after two 5-min washes in PBS. Sections were incubated at 4 °C overnight with the mouse monoclonal anti-chicken chemerin antibody diluted 1:200 in PBS-Bovine Serum Albumin (BSA). After washes in PBS—0.05% Tween 20 and PBS, slides were incubated in goat anti-mouse Alexa 488 (diluted at 1:500 in PBS for 1 h). After 3 5-min washes in PBS, sections were mounted with Vectashield. Negative controls were performed by replacing antibody with rabbit IgG (Sigma-Aldrich, l’Isle d’Abeau Chesnes, France). Immunostaining was performed on five tissue sections from six different animals.

### Antibacterial activity test

The antibacterial activity of chemerin was investigated against three pathogenic bacterial strains commonly used for quality control in antibiotic susceptibility testing, which included two Gram-negative bacteria (*Escherichia coli* Collection Institut Pasteur (CIP) 7624/ATCC 25922/DSM 1103/CCUG 17620/CECT 434/NCTC 12241 and *Pseudomonas aeruginosa* CIP 76110/ATCC 27853/NCTC 12903/DSM 1117/CCUG 17619/CECT 108) and one Gram-positive bacterium (*Staphylococcus aureus* CIP 7625/ATCC 25923/CCM 3953/CECT 435/CCUG 17621/DSM 1104/NCTC 12981). All bacterial strains used in this study were obtained from the “Collection de l’Institut Pasteur” (CIP) Paris, France. The determination of the recombinant chicken chemerin potency against bacterial strains was made by assessment of its minimal inhibitory concentrations (MIC), using a standardised broth microdilution assay procedure (CASF/EUCAST 2019). Strains stored at − 80 °C in Brain Heart Infusion (BHI) broth (BD Difco) glycerol 15%, v/v (Carlo Erba)). They were first incubated overnight in 5 mL of BHI broth, and subsequently, BHI agar plates were streaked with the overnight cultures and incubated for 24 h. Few isolated colonies were emulsified in sterile saline solution until matching the turbidity standard 0.5 McFarland (1–5.10^8^ CFU/mL). Inocula were prepared by diluting 0.5 McFarland suspension 10 times in Mueller Hinton Broth (MHB, BD Difco).

Recombinant chicken chemerin at 0.5 mg/mL (20 mM sodium phosphate, 250 mM sodium chloride buffer, pH 7) was twofold serially diluted in MHB in a 100-well sterile honeycomb microplate (100 µL of diluted chemerin/well). Then, 100 µL of bacterial inoculum was added to obtain final chemerin concentrations of 125, 62.5, 31.25, 15.63, 7.8, 3.9, 1.95, and 0.97 µg/mL for a final concentration of about 10^5^ CFU/mL of bacteria.

A dilution range of the buffer used to prepare recombinant chicken chemerin (20 mM sodium phosphate, 250 mM NaCl, pH 7), identical to that of recombinant chemerin (1/4–1/512), was carried out to assess the impact of salts on the growth of strains. The sterility of the culture medium, the buffer and chemerin was checked in each test. The microplate was incubated at 37 °C in an automatic growth curves analyser (Bioscreen C MBR, Thermo Fisher Scientific, Saint-Herblain, France). The optical density (OD) at 600 nm was measured every 45 min for 24 h (shaking before each measurement with following parameters: high, normal, 10 s). Experiments were repeated twice. The MIC was defined as the lowest concentration of chemerin showing no visible growth (flat curve) after 24 h.

### Chorio allantoic membrane (CAM) assay

The CAM assay was performed according to Storgard et al.^29^. Briefly, since the allantois of the chick embryo appears at about 3.5 days of incubation, eggs from local breeders were artificially incubated for 10 days. (37.5 °C, relative humidity: 55–65%). On the 10th incubation day, a false air chamber was created directly over the CAM, permitting its detachment from the shell membrane. To decrease the risk of infection, the whole eggs were cleaned and sterilised with 70% ethanol. The procedure was continued through a square incision of the eggshell over the CAM. The prepared window in a square form (2 cm^2^), was covered by a flexible film and then transported to the incubator. One hour after window opening, a paper disc (6 mm in diameter) (Whatman) was coated with target agents diluted in a total volume of 10 μL of PBS (0.1 and 1 μg anti-chemerin antibody, and 0.1 and 1 μg anti-CMKLR1 antibody) and placed in the central area of the corresponding window. The CAM surface was visually evaluated after 72 h of additional incubation using a stereomicroscope. We decided to use the surface of capillary blood vessels as the index of anti-angiogenic activity. Consequently, the prepared images were analysed using the Image J software to determine the percentage of the surface of the paper disc occupied by blood vessels.

### Statistical analysis

The GraphPad Prism^®^ software (version 8) was used for all analyses. Data were tested for homogeneity of variance by Bartlett's test, and for normal distribution by the Shapiro–Wilk test. A one-way ANOVA was performed with Tukey–Kramer multiple comparisons tests, or with Dunnett's to compare controls, as appropriate. Mann–Whitney tests were performed where variances were unequal. Culture data included replicates as a random variable. Chemerin amounts in blood plasma compared to albumen were analysed by the unpaired t-test. Data are presented as means ± SEM, with p < 0.05 considered significant.

## Results

### Expression of chemerin within the egg

As shown in Fig. 1A, chemerin protein as determined by immunoblot is highly abundant in the perivitelline membranes and the white (albumen) as compared to the egg yolk. Samples of the hens' blood plasma and albumen were also analysed. As shown in Fig. 1B, the amount of chemerin was about 10 times higher in the albumen compared to the plasma. Similar data were obtained by the ELISA assay (data not shown). However, the chemerin protein level was similar in thin and thick albumen but more abundant in the chalaza (Fig. 1C). By immunofluorescence, we confirmed the presence of chemerin inside the perivitelline membrane, with a significant staining of this structure when incubated with an anti-chemerin antibody, whereas there was no staining with diamidino-2-phenylindol (DAPI) since this structure is acellular (Fig. 1D).

### Chemerin in egg white comes from magnum

Since the albumen is produced and secreted during egg formation by the oviduct in hens, we investigated the expression of chemerin and its three receptors within the five parts of the hen oviduct (infundibulum, magnum, isthmus, shell gland and vagina, Fig. 2A). As shown in Fig. 2B, *RARRES2* gene expression, as determined by RTqPCR was significantly higher in both infundibulum and magnum compared to the isthmus, shell gland, and vagina. By immunoblot (Fig. 2C) and immunofluorescence (Fig. 2D) we confirmed these data for the magnum but not for the infundibulum. Using in vitro culturing of magnum explants from laying hens, we demonstrated that magnum explants can secrete chemerin in the culture medium as measured by an ELISA assay (Fig. 2E). Moreover, hormonal stimulation of explants with both E2) (10^–8^ M, 48 h) and P4 (10^–8^ M, 48 h) significantly increased chemerin secretion in the conditioned culture medium (Fig. 2E) and the chemerin protein level in the magnum explants (Fig. 2F).

**Figure 3:**
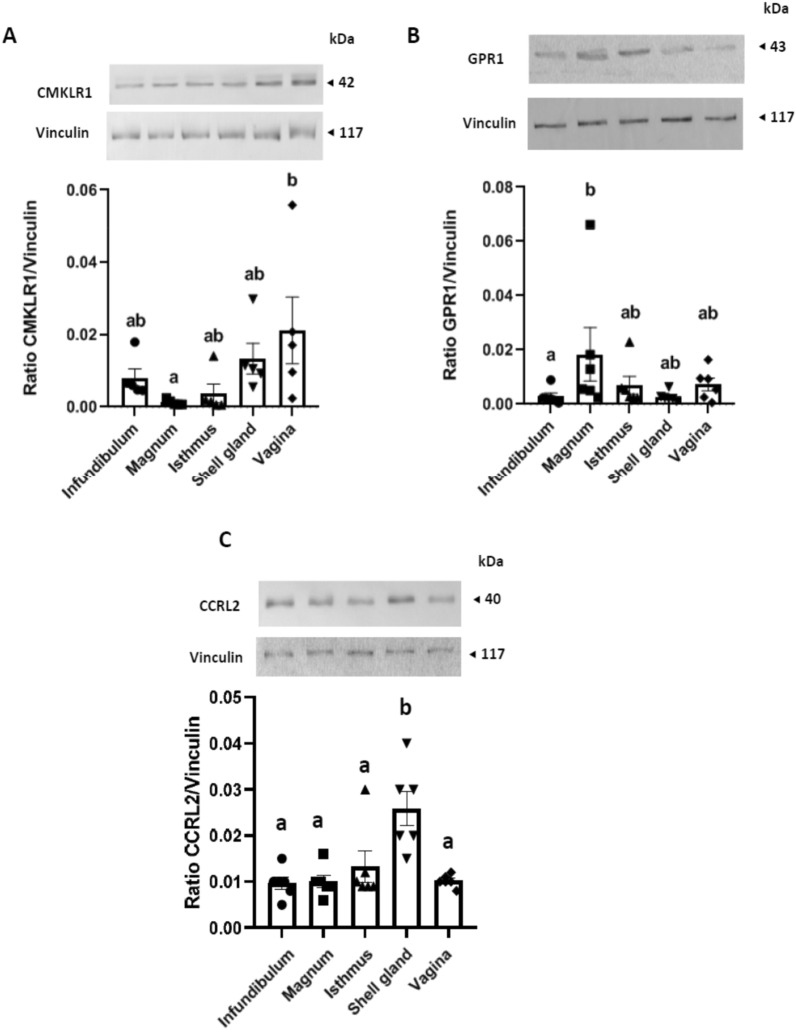
Chemerin receptor protein expression in the laying hen oviduct. Protein abundance of Chemokine-like receptor 1 (CMKLR1) (**A**), G Protein-coupled Receptor 1 (GPR1) (**B**), and Chemokine (C–C motif) receptor-like 2 (CCRL2) (**C**) detected by western-blotting within the infundibulum, magnum, isthmus, shell gland and vagina of hens (n = 6). Different letters indicate significant differences at p < 0.05.

We also used a western blot to show that the three chemerin receptors, CMKLR1, GPR1 and CCRL2 are found in hen oviducts with a maximum expression in the vagina, magnum and shell gland, respectively (Fig. 3A–C).

### Chicken chemerin antibacterial activity

The antibacterial activity of recombinant chicken chemerin was investigated through the determination of its minimal inhibitory concentrations (MIC), using a standardised broth microdilution assay procedure (CASFM/EUCAST 2019). The MICs of recombinant chicken chemerin obtained after 24 h at 37 °C were 125 µg/mL for each tested bacterial strain of *Escherichia coli*, *Staphylococcus aureus* and *Pseudomonas aeruginosa*. A partial inhibition of bacterial growth, which resulted in a delayed growth, was still observed at the concentration of 62.5 µg/mL of recombinant chicken chemerin (data not shown). The effect of buffer salts alone, diluted in the same way as the recombinant chemerin, was checked. Buffer salts slightly reduced the bacterial growth by 19.5% ± 2.5% for *P. aeruginosa*, 16% ± 1% for *Escherichia coli* and 7.5% ± 1.5% for *Staphylococcus aureus,* depending on the assay when considering the maximum optic density decrease between growth control curve points in MHB and growth curve points in MHB/buffer (data not shown).

### Chemerin system expression in embryo annexes and fluids

**Figure 4:**
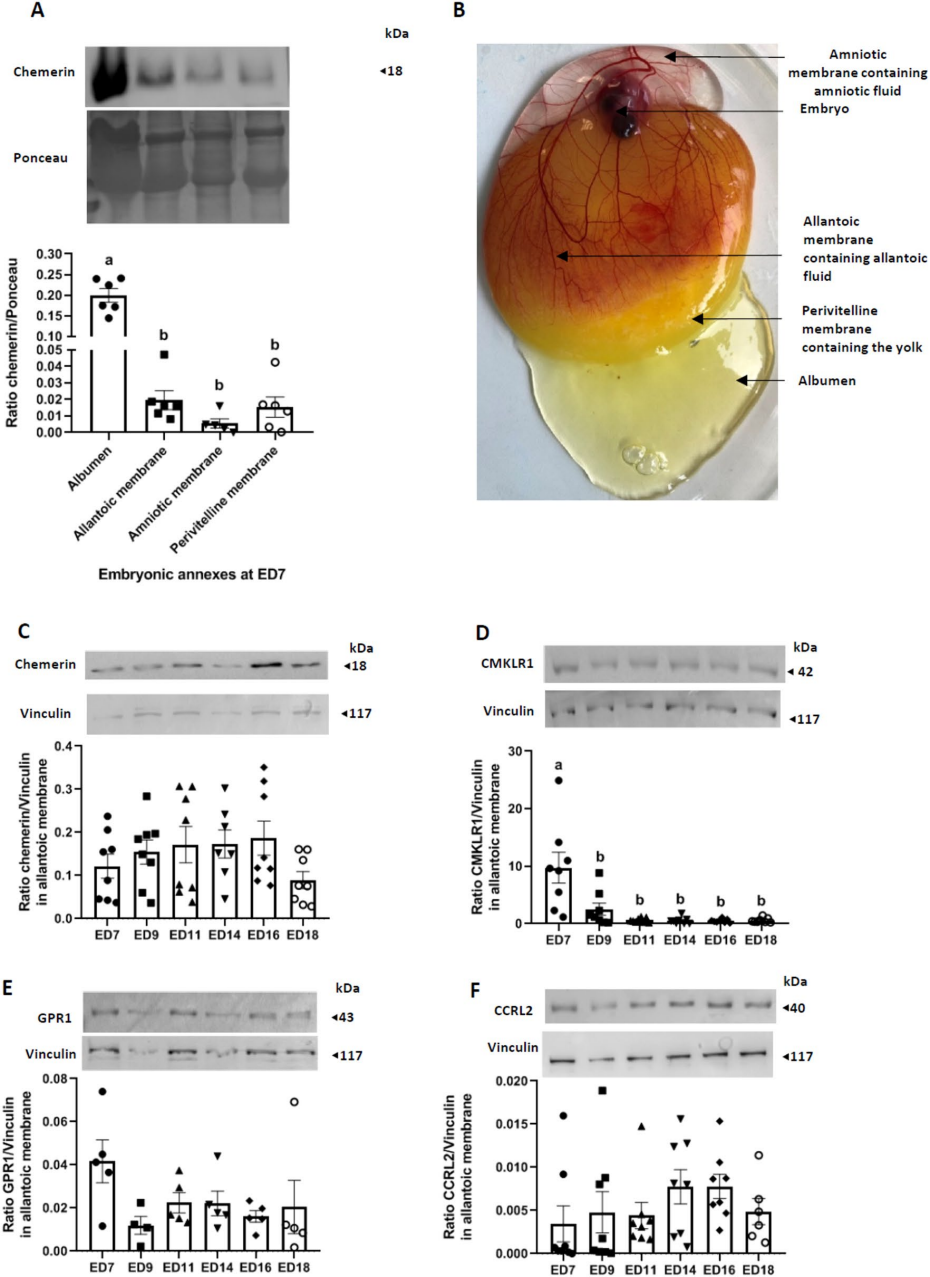
Chemerin system expression in embryo annexes and fluids. (**A**) Protein abundance of chemerin detected by western blotting within the albumen (thin and thick), allantoic membrane, amniotic membrane, and the perivitelline membrane from fertilised eggs incubated for 7 days (n = 6). The ratio chemerin/ponceau is represented. (**B**) Localisation of the different embryonic structures at ED7. (**C**–**E**) Protein abundance of chemerin (**C**), Chemokine-like receptor 1 (CMKLR1) (**D**), G Protein-coupled Receptor 1 (GPR1) (**E**) and Chemokine (C–C motif) receptor-like 2 (CCRL2) (**F**) detected by western blotting within the allantoic membrane of incubated eggs at different embryonic days (ED): 7, 9, 11, 14, 16, and 18 (n = 8 per stage). Values are expressed as mean ± standard errors of means. Letters indicate significant differences between various conditions (p < 0.05).

Since chemerin is present in the egg white, we determined whether this hormone could be involved in embryogenesis. First, we collected samples of embryonic annexes (allantoic and amniotic membranes) and amniotic fluids from eggs artificially incubated for 7 days (Fig. 4B). Western blot data showed that chemerin is found in the albumen and the perivitelline membranes of the eggs, as previously shown in unincubated eggs, but also expressed by the allantoic and amniotic membranes (Fig. 4A). We measured the expression of chemerin and its receptors by the allantoic and amniotic membranes at different embryonic days (ED): ED 7, 9, 11, 14, 16 and 18. In the allantoic membranes, the amount of chemerin (Fig. 4C), GPR1 (Fig. 4E) and CCRL2 (Fig. 4F) remained stable from ED7 to ED18, whereas for CMKLR1 we found maximum protein expression at ED7, followed by a significantly reduced and stable expression for the remaining the incubation period (Fig. 4D).

**Figure 5:**
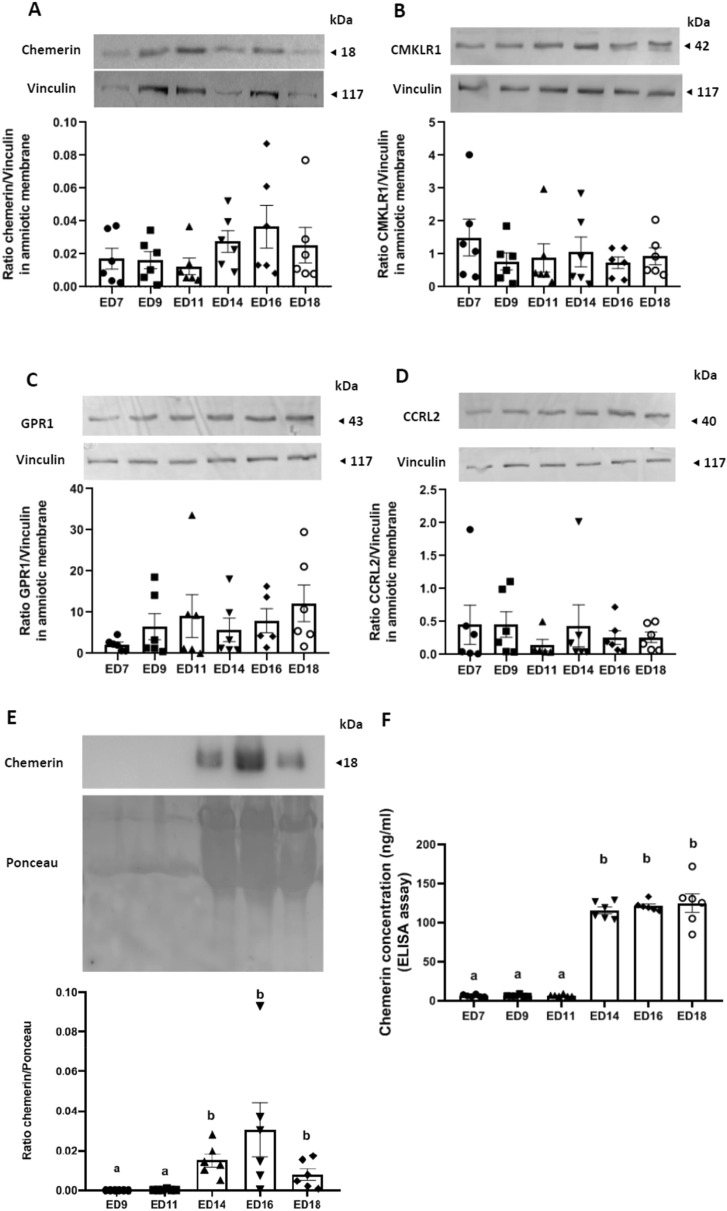
Chemerin system expression in amniotic membrane and fluid. (**A**–**D**) Protein abundance of chemerin (**A**), Chemokine-like receptor 1 (CMKLR1) (**B**), G Protein-coupled Receptor 1 (GPR1) (**C)** and Chemokine (C–C motif) receptor-like 2 (CCRL2) (**D**) detected by western blotting within the amniotic membrane of incubated eggs at different embryonic days (ED): 7, 9, 11, 14, 16 and 18 (n = 8). (**E**) Protein abundance of chemerin detected by western blotting within the amniotic fluid of incubated eggs at embryonic days (ED) 9, 11, 14 16 and 18 (n = 8). (**F**) Chemerin concentrations measured by Enzyme Linked Immunosorbent Assay in the amniotic fluids of incubated eggs at embryonic days (ED) 7, 9, 11, 14, 16 and 18 (n = 8). Values are expressed as mean ± standard errors of means. Letters indicate significant differences between different conditions (p < 0.05).

For the amniotic membranes, the protein amount of chemerin (Fig. 5A) and its three receptors, CMKLR1 (Fig. 5B), GPR1 (Fig. 5C) and CCRL2 (Fig. 5D) remained stable for the entire incubation period. By western-blotting (Fig. 5E) and the ELISA assay (Fig. 5F), we showed that the amount of chemerin in the amniotic fluid was extremely low from ED9 to ED11 and then significantly increased from ED14 and remained high until ED18. In contrast, the amount of chemerin in the allantoic fluid was unchanged during the entire incubation period studied (data not shown).

### In-ovo injections of neutralizing chicken chemerin and CMKLR1 antibodies increase embryo mortality

**Figure 6:**
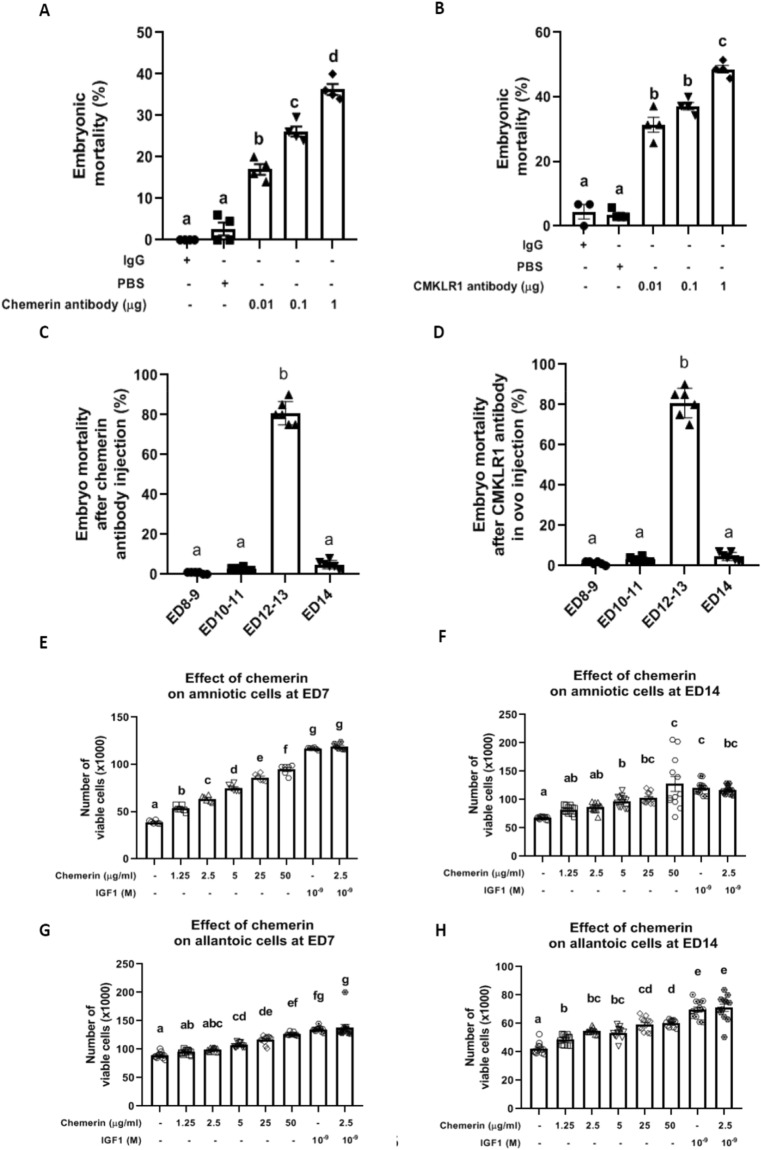
Role of chemerin during embryo development and effect of recombinant chicken chemerin on the viability of amniotic and allantoic cells, measured using the Cell Counting Kit-8 (CCK8) assay at ED7 and ED14*.* (**A**,**B**) Percentage of embryonic mortality of fertilised eggs injected at ED7 with 100 μL of Immunoglobulin G, Phosphate Buffer Saline and different amounts of anti-chemerin (**A**) and anti- Chemokine-like receptor 1 (CMKLR1) (**B**) antibodies (0.01, 0.1, and 1 μg). Embryo mortality was measured daily by candling and breaking eggs. (**C**,**D**) Repartition of the percentage of embryo mortality according to the day of embryo development (ED): ED8–9, ED10–11, ED12–13 and ED14. Values are expressed as mean ± standard errors of means. Letters indicate significant differences between different conditions (p < 0.05). (**E–H**) Effect of recombinant chicken chemerin on the viability of amniotic (**E–F**) and allantoic (**G**–**H**) cells, measured using the CCK8 assay at ED7 (**E–G**) and ED14 (**F–H**). Amniotic and allantoic cells were incubated with different concentrations of recombinant chicken chemerin (1.25, 2.5, 5, 25, and 50 µg/mL) for 24 h or with IGF1 alone as positive control, or combined recombinant chicken chemerin at 2.5 µg/mL. Values are expressed as mean ± standard errors of means. Letters indicate significant differences between conditions (p < 0.05).

Subsequently, we investigated the role of chemerin during embryo development by in-ovo injections of neutralising chicken antibodies directed against either chemerin or CMKLR1 in albumen at ED7. Once injected, the eggs were artificially incubated for 1 week and candled daily. As shown in Fig. 6A, eggs injected with IgG had a low rate of embryonic mortality (1–3%) from ED7 to ED14. This rate was similar to that of incubated PBS-injected fertilised eggs. Eggs injected with 0.01, 0.1, and 1 μg of anti-chemerin antibodies showed a significant increase in embryonic mortality around 17 ± 1.3% (p < 0.0001), 26.15 ± 1.2% (p < 0.0001) and 36.25 ± 1.3% (p < 0.0001) compared to eggs injected with IgG or PBS, respectively. Similarly, in-ovo injections of 0.01, 0.1, and 1 μg of anti-CMKLR1 antibodies significantly increased embryonic mortality to 31.4 ± 2.3% (p < 0.0001), 37.13 ± 1.2% (p < 0.0001) and 48.58 ± 1.2% (p < 0.0001), respectively (Fig. 6B). After candling and breaking eggs, we observed that more than 80% of embryo mortality after anti-chemerin or anti-CMKLR1 antibodies in-ovo injection occurred at ED12-13 (Fig. 6C,D**)**.

### Chicken recombinant chemerin increases in-vitro amniotic and allantoic cell proliferation

As shown in Fig. 6E–H, we also determined the effect of recombinant chicken chemerin on the growth of primary amniotic (Fig. 6E–F) and allantoic cells (Fig. 6G–H) recovered from incubated eggs at ED7 and ED14. Recombinant chicken chemerin treatment (1.25, 2.5, 5, 25 and 50 μg/mL) increased in a dose-dependent manner *in-vitro* proliferation of amniotic (Fig. 6E,F) and allantoic cells (Fig. 6G,H) at both ED7 and ED14 (p < 0.0001). However, at the 2.5 μg/mL concentration, it did not affect the stimulatory effect of IGF-1 on the proliferation of these two chicken cell types (Fig. 6E–H**)**.

### Involvement of the chemerin in angiogenesis within the chorioallantoic membrane

**Figure 7:**
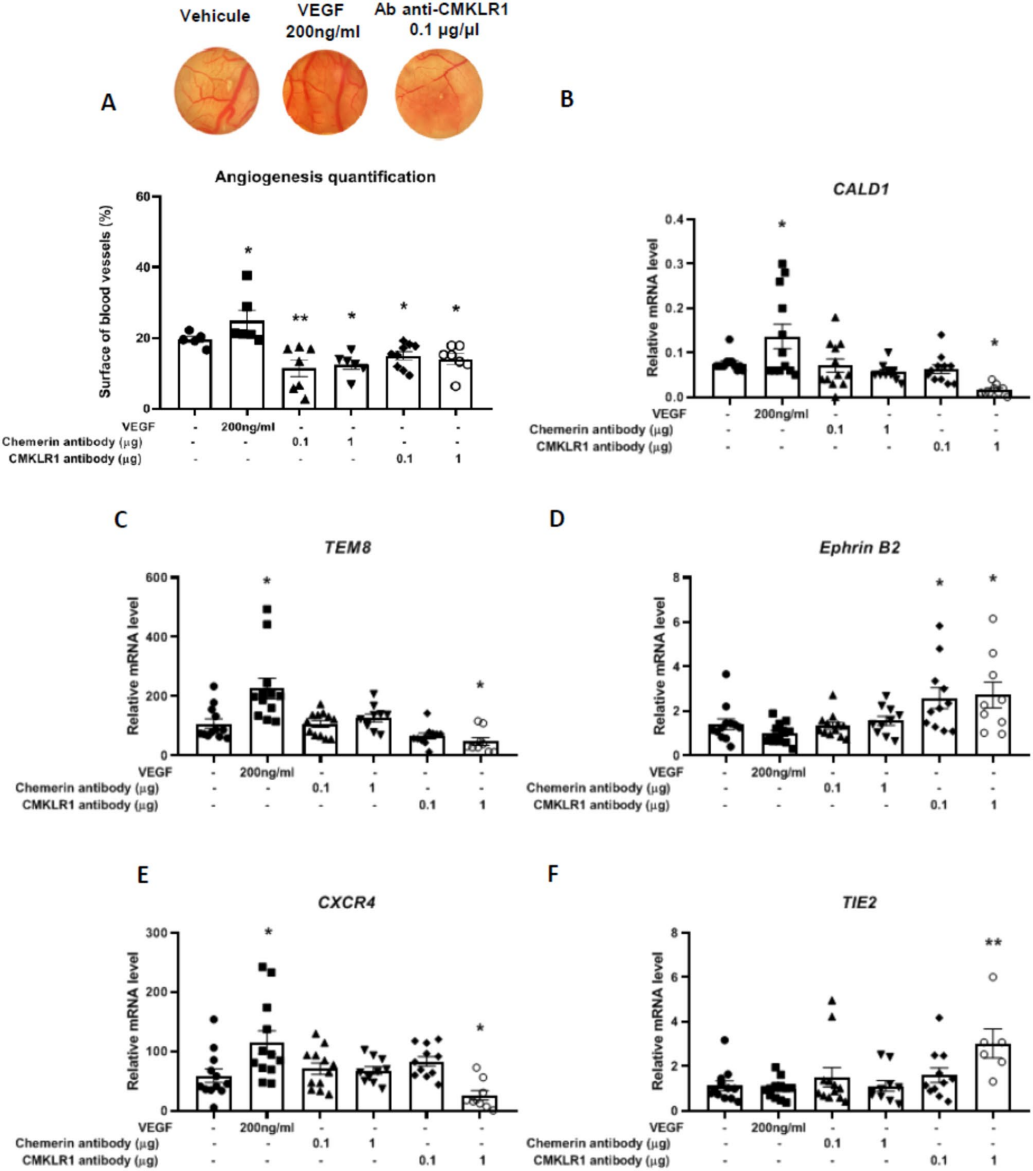
Involvement of recombinant chicken chemerin in angiogenesis within the chorioallantoic membrane. (**A**) Angiogenesis quantification of the chorioallantoic membrane (CAM) when treated with vehicle, Vascular Endothelial Growth Factor (VEGF) (200 ng/mL), anti-chemerin antibodies (0.1 and 1 μg/μL) and anti- Chemokine-like receptor 1 (CMKLR1) antibodies (0.1 and 1 μg/μL). After 72 h of local treatment of the CAM with a coated Whatman paper disc, different treatments/doses were deposited on the surface of the CAM, and the membranes were fixed with 4% Paraformaldehyde (PAF), sampled and photographed under a stereoscopic microscope. The surface of the disc occupied by blood vessels was measured with the ImageJ software, and results are expressed in percentage of blood vessels on the total surface of the membrane under the Whatman paper disc (n = 8 assays per treatment). (**B**–**F**) Relative mRNA levels of Caldesmon 1 (CALD1) (**B**), Tumour Endothelial Marker 8 (TEM8) (**C**), EPHRIN B2 (**D**), C-X-C motif chemokine receptor 4 (CXCR4) (**E**) and Tyrosine kinase with Immunoglobulin and EGF homology domains 2 (TIE2) (**F**) measured by RT-qPCR (n = 8 assays per treatment). Values are expressed as mean ± standard errors of means. *Indicates significant differences between controls and treated CAM (*p < 0.05, ** p ˂ 0.01).

Subsequently, we determined the role of chemerin and CMKLR1 in angiogenesis by using the ChorioAllantoic Membrane assay (CAM assay) of fertilised chicken eggs (Fig. 7A). After topical application of neutralising anti-chemerin antibodies at 0.1 and 1 μg and incubation for 72 h, the CAM clearly showed a reduced percentage of the vascularised surface (11.5% ± 2.3% (p = 0.0009) and 12.5% ± 1.3% (p = 0.0028), respectively) when compared with PBS-treated eggs (19.5% ± 0.9%). Similarly, eggs treated with anti-CMKLR1 antibodies at 0.1 and 1 µg showed a significant reduction in the percentage of the surface of the vascularised area as compared to the control at 15% ± 1.2% (p = 0.0219) and 14% ± 1.5% (p = 0.0178), respectively (Fig. 7A). In contrast, eggs that had received an absorbent paper disc imbibed with Vascular Endothelial Growth Factor (VEGF) at 200 ng/mL (positive control) showed a significant increase in the percentage of the vascularised surface at almost 25% ± 2.9% (p = 0.0138) (Fig. 7A).

To better understand the effect of the chemerin system on the angiogenesis of chicken embryos, we analysed, by RTqPCR, the CAM expression of several genes involved in angiogenesis. As shown in Fig. 7B,C,E, we observed an increase in the expression of specific markers such as *Caldesmon 1* (*CALD1*) (p = 0.0188), *Tumour Endothelial Marker 8 (TEM8)* (p = 0.0001) and *C-X-C motif chemokine receptor 4* (*CXCR4)* (p = 0.0048) when CAM was treated with the pro-angiogenic VEGF at 200 ng/mL. On the other hand, when CAM was treated with 1 μg of the neutralising anti-CMKLR1 antibody, the expression of the same genes was significantly decreased when compared to the control condition (p = 0.0407 for *CALD1*; p = 0.0139 for *TEM8*; p = 0.028 for *CXCR4*). For samples of CAM treated with 0.1 and 1 μg of neutralizing anti-chemerin antibody and 0.1 μg of anti-CMKLR1 antibody, we did not find any significant differences in gene expression (Fig. 7B,C,E). Unlike the previously studied genes, we observed a significant increase in *EPHRIN B2* (p = 0.0392 for 0.1 μg and p = 0.0260 for 1 μg) and *Tyrosine kinase with Immunoglobulin and EGF homology domain 2* (*TIE2)* (p = 0.0027) gene expression when CAM was treated with 1 μg of neutralising anti-CMKLR1 antibody and 0.1 and 1 μg of the same antibody, respectively (Fig. 7D,F).

## Discussion

The present study is the first to show that an adipokine, here chemerin, is strongly expressed in the magnum, a segment of hen oviduct, and is consequently found in the egg white. We also observed that chemerin secretion was in vitro induced by a treatment composed of P4 plus E2 on magnum explants. Moreover, we demonstrated that chemerin and its three receptors, CMKLR1, GPR1 and CCRL2, were present in allantoic and amniotic membranes, and the amount of CMKLR1 in the amniotic membranes decreased from ED7 to ED14 and remained extremely low until ED18. Adversely, we detected a strong abundance of chemerin in amniotic liquid at ED14. Finally, after in-ovo injections of neutralising anti-chemerin or anti-CMKLR1 antibodies at ED7, we revealed an increase in embryo mortality at ED14. These data were associated to a reduction in the gene expression of angiogenic markers when CAMs are locally treated with anti-CMKLR1 antibody. Taken together, our findings suggest that chemerin from albumen, through its passage in the amniotic fluid, could exert a beneficial role in chicken embryogenesis.

So far, there is very little information about the role of chemerin in reproductive functions in birds. Most data are derived from studies on chickens and turkeys and are related to the link between the metabolism and the reproductive function^20–23,30^. Our laboratory has already demonstrated that in chickens, plasma chemerin concentrations are negatively correlated with egg hatchability, suggesting a potential role of this adipokine on egg^22^. Here, we demonstrate that chemerin is 10 times less concentrated in plasma than in egg albumen. In addition, chemerin is found abundantly in the perivitelline membrane. These observations raise the question about the role of chemerin, locally produced within the oviduct, on egg fertility or on its potential role in embryonic development. The main sources of chemerin production are the white adipose tissue and the liver^1^. In hens, the liver synthesises and secretes yolk precursors that are incorporated into the developing yolky follicles by endocytosis. Yolk precursors are transported to the follicular wall via the blood stream^31^. However, our results demonstrate that chemerin is mainly found within the albumen of the egg, produced by the hen oviduct, and not the yolk, which contains undetectable concentrations of this hormone. We also suggest that the chemerin accumulation in the egg is due to its secretion by the infundibulum and/or the magnum of the hen oviduct, giving it a local origin within the tract and a lower amount in the blood plasma. Indeed, we demonstrated that chemerin expression at mRNA and protein levels, is mainly found in these parts of the oviduct. Our results are in agreement with a previous study demonstrating that chemerin is expressed by the oviductal magnum^32^.

We also found that chemerin is abundant in the perivitelline membrane that surrounds the egg yolk. In mammals, this perivitelline membrane composed of insoluble extracellular matrix is called “zona pellucida” (ZP)^33^. Until now, chemerin has never been reported in the ZP. Mann et al.^34^ also identified chemerin [similar to the retinoic acid receptor responder (tazarotene-induced) 2 (TIG-2)] by a proteomic analysis in the chicken egg vitelline membrane^34^. In chickens, the perivitelline membrane is composed of two layers, an inner layer, deposited in the preovulatory phase, and an outer layer, added during passage through the oviduct; these layers are separated by a thin membrane^35,36^. This membrane and the outer layer are added to the inner membrane only after ovulation*,* (i.e., during migration of the oocyte through the oviduct)^37,38^. For successful fertilisation to occur, sperm first has to bind to the perivitelline membrane, and then penetrate it^39^. The outer layer appears to be involved in to the blocking of polyspermy via the acrosome reaction^40^. Thus, chemerin could participate in the fertilisation process in chickens as well. The physiological functions of the perivitelline membrane are not only related to fertilisation and early stages of embryogenesis, but also to the physical and molecular protection of the embryo.

Our results provide more evidence of a putative role of chemerin in embryo development, based on the increase of embryonic death when the chemerin system is inhibited by in-ovo injections of anti-chemerin and anti-CMKLR1 antibodies. We found that most of the embryos died at ED 12–13, the period when we detected a strong increase of chemerin in the amniotic fluid. In addition, we showed the presence of the chemerin system within the embryonic annexes and fluids at different stages of incubation. In amniotic fluid, we detected a high amount of chemerin at ED14 as compared to ED11. This can be explained by the transfer of egg white in the amniotic sac, which has been well studied during the development in the chicken *Gallus gallus*^41–43^. Indeed, after ED12, egg white proteins are transferred in mass into the amniotic sac^44,45^, where they are absorbed orally by the embryo as a source of amino acids to support its rapid growth^46^ until hatching (ED21). Egg white is also known for its role in the defense of the embryo by its high concentration of antimicrobial proteins^47^; as for the vitelline membrane, it also contains antimicrobial proteins to form a last protective barrier. This raises the question of whether chemerin does not play a protective role within the egg, since it is therefore found in two regions involved in the defense of the egg. In addition, human chemerin has been described to have antimicrobial activities^48^. Despite the presence of salts, playing probably a minor role in the measured antibacterial effects of recombinant chicken chemerin, our results suggest that chicken chemerin can also inhibit the growth of several bacterial species. In comparison, the MIC (Maximal Inhibitory Concentration) values obtained by Banas et al.^48^, with human chemerin-derived synthetic peptide (p4), were 3.1–6.3 µg/mL for *E. coli*, 12.5 µg/mL for *S. aureus* and 6.3 µg/mL for *P. aeruginosa*. These MIC values are much lower than those we determined for recombinant chicken chemerin (125 μg/mL), but the protocol used by Banas et al.^48^ is largely different from what was done in the present study. The use of poor nutrient medium [10 mM sodium phosphate buffer pH 7.4 containing only 1% (v/v) trypticase soy broth (TSB)], as well as the use of a low bacterial inoculum, could indeed favour the antibacterial effect of human chemerin. Moreover, the physiological state of the inoculum used may influence its susceptibility to chemerin as Godlewska et al.^49^ reported that the lethal effects of chemerin are enhanced by bacterial-derived Reactive Oxygen Species-induced chemerin peptide oxidation and suppressed by stationary phase sigma factor, RpoS. Moreover, the MIC results obtained by Banas et al.^48^ were those of the domain Val66-Pro85 peptide (p4), localised in the middle of the chemerin sequence, which expresses the majority of chemerin’s anti-microbial activity^48^. The removal of a terminal inhibitory peptide of the recombinant chicken protein could thus allow antibacterial activity at lower concentrations.

A key defining characteristic of amniotes (mammals, reptiles, and birds) is the formation of four extraembryonic membranes during embryonic development: the amnion, chorion, allantois, and yolk sac^50^. Fusion of the chorion and allantois forms either the chorioallantoic placenta in viviparous (live-bearing) species, or the chorioallantoic membrane (CAM) in oviparous (egg-laying) species^51^. Both the chorioallantoic placenta and CAM perform functions crucial for embryonic survival and development^50,51^. In mammals, chemerin acts as a chemoattractant and has been suggested to play a role in placentation by regulating Natural Killer cell accumulation and endothelial cell morphogenesis during early pregnancy^19,52^. In mice and humans, the chemerin/GPR1 system is expressed in the placenta, with a putative role as a feedback mechanism that could regulate the carbohydrate balance during pregnancy^53^. Yang et al.^54^ showed that intrauterine injection of a CMKLR1 receptor antagonist in pregnant mice results in abortion, suggesting a potential role of chemerin/CMKLR1 signalling in the maintenance of pregnancy. In women, patients with early abortions have lower levels of chemerin expression at the decidual cell level than those with a non-problematic pregnancy^54^. Therefore, it seems that in chickens, rodents and humans, the chemerin/CMKLR1 system plays a crucial role in embryonic development. In chickens, we showed that chemerin and its receptors are expressed in CAM. In humans, chemerin is also known as a pro-inflammatory adipokine, induced by Tumor Necrosis Factor (TNF)-alpha and enhancing macrophage adhesion^55^, as well as a potent angiogenic factor, inducing the gelatinolytic activity of endothelial cells^56–58^. The latter observation is in accordance with our results concerning the deleterious effect of chemerin system inhibition on the vascularisation of the CAM and on the associated variation gene marker expression in chickens. Indeed, we noticed an increase in *CALD1*, *TEM8,* and *CXCR4* expression in CAM samples when stimulated by the pro-angiogenic factor, VEGF, and a decrease of their expression levels when the chemerin system is inhibited by anti-CMKLR1 antibodies at the highest dose. In contrary, we observed a significant increase of *EPHRIN B2* and *TIE 2* in CAM treated with the same antibody. These results are in accordance with observations showing that *CALD1*, *TEM8,* and *CXCR4* are increased during proangiogenic development within the CAM, whereas *EPHRIN B2* and *TIE 2* decrease in the same context^59,60^, underlying an active angiogenesis activity of chicken chemerin.

In conclusion, we showed, for the first time, that a maternal adipokine, chemerin, produced by the hen oviduct, accumulates in albumen and could be involved in early embryonic development by promoting normal metabolism of the embryo. Moreover, we also demonstrate that chemerin is important in the normal functions of the annexes, such as chorio-allantoic membrane angiogenesis, necessary for embryo development, and in egg protection against bacterial development. Since fertility and hatchability are the main indicators of reproductive output in chickens, it is highly important to better understand and study ways to improve these parameters. Indeed, in poultry farming enterprises, even small variations in fertility and hatchability rates could have huge financial repercussions due to the large volume of animals generated. Chemerin seems to be a key component of the egg and, more specifically, of the albumen, due to its role in supporting the development of the embryo and the annexes as well as its antibacterial properties. Thus, we are conducting further research to highlight whether the concentration of chemerin in egg white could be a good predictive marker of egg hatch and serve as a tool to select good breeders in poultry farming.

## Supplementary Information

Supplementary Figures.

## Data Availability

Some or all data generated or analyzed during this study are included in this published article or in the data repositories listed in References.
