# Supplementary material for: Chemerin is secreted by the chicken oviduct, accumulates in egg albumen and could promote embryo development
Source: Sci Rep. 2022 May 29;12:8989. doi: 10.1038/s41598-022-12961-4 (PMC9148909; doi:10.1038/s41598-022-12961-4)

Figure 1A

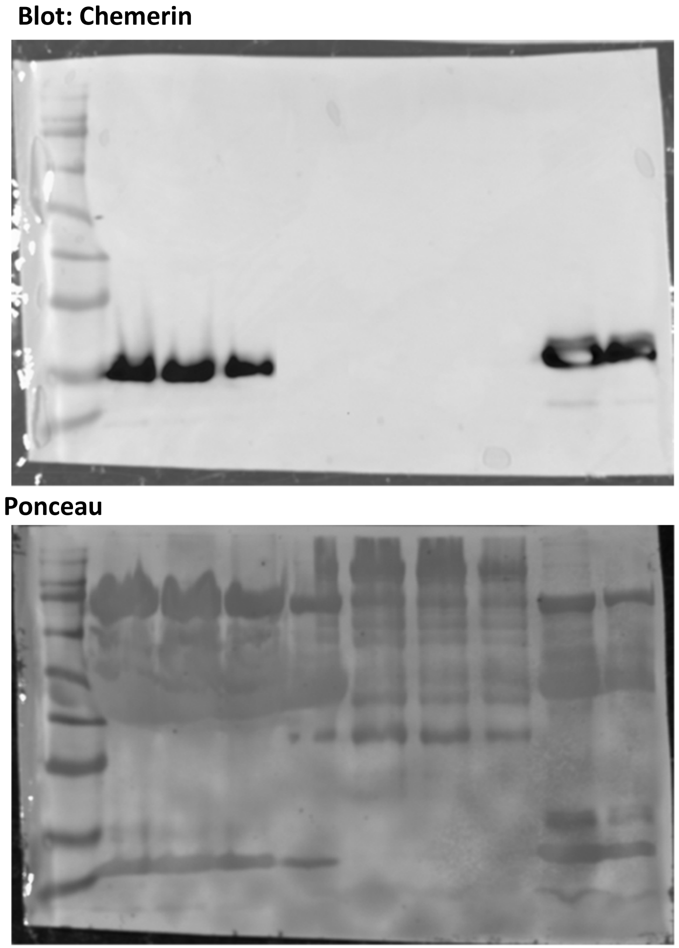

Figure 1B

Standard molecular Weight:

Ref: BlueStar Prestained Protein Marker11

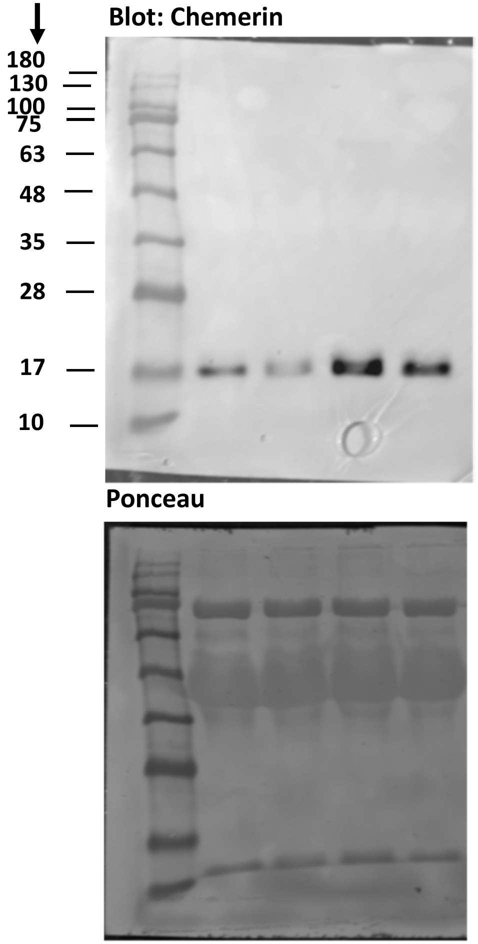

Figure 1C

Standard molecular Weight:

Ref: SDS7B2 (Sigma Aldrich)

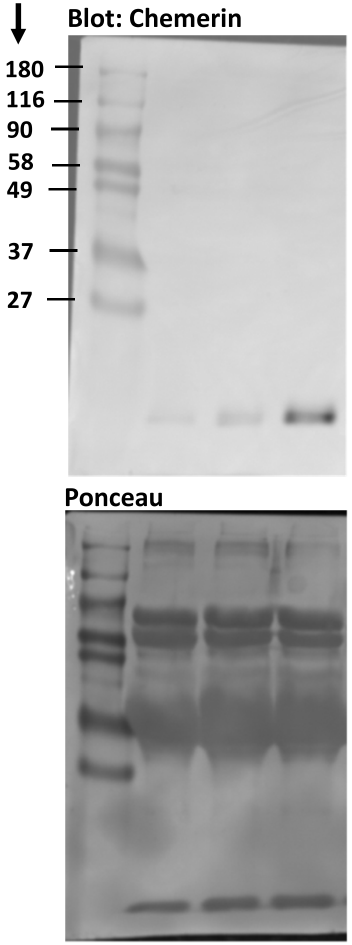

Figure 2C

Blot: Chemerin

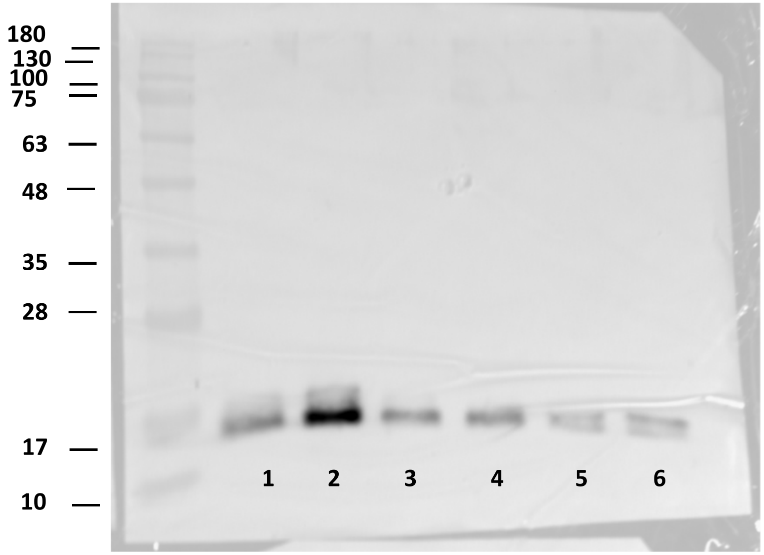

1: Infundibulum; 2: Magnum; 3: Utero-vaginal junction; 4: Isthmus; 5: Shell gland; 6: Vagina

In the figure 2C in our manuscript we removed the Utero-vaginal junction

Blot: Vinculin

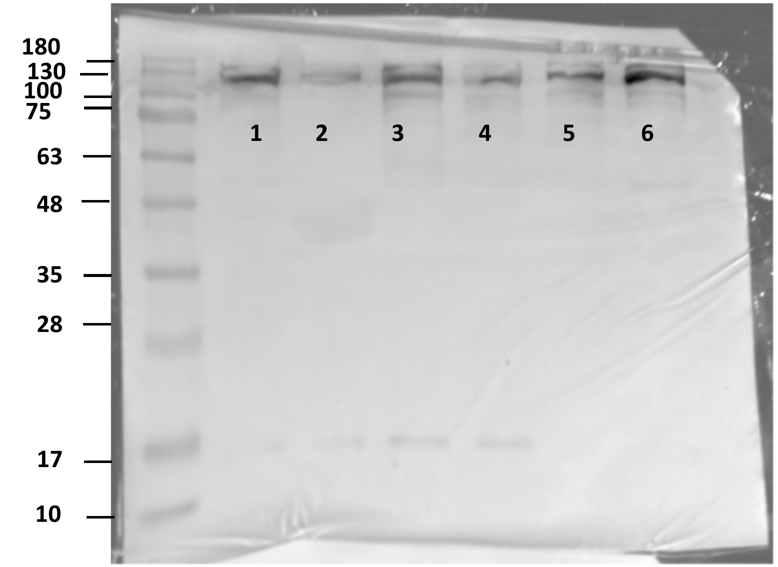

1: Infundibulum; 2: Magnum; 3: Utero-vaginal junction; 4: Isthmus; 5: Shell gland; 6: Vagina

In the figure 2C in our manuscript we removed the Utero-vaginal junction

Figure 2F

Blot: Chemerin

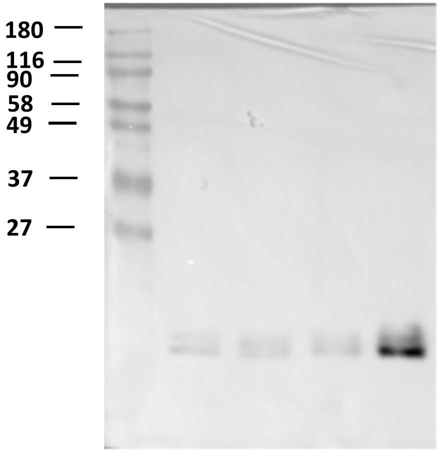

Blot: Vinculin

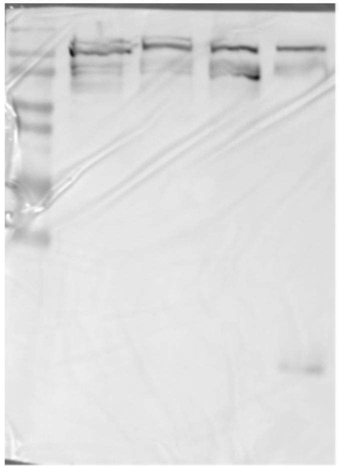

Figure 3A

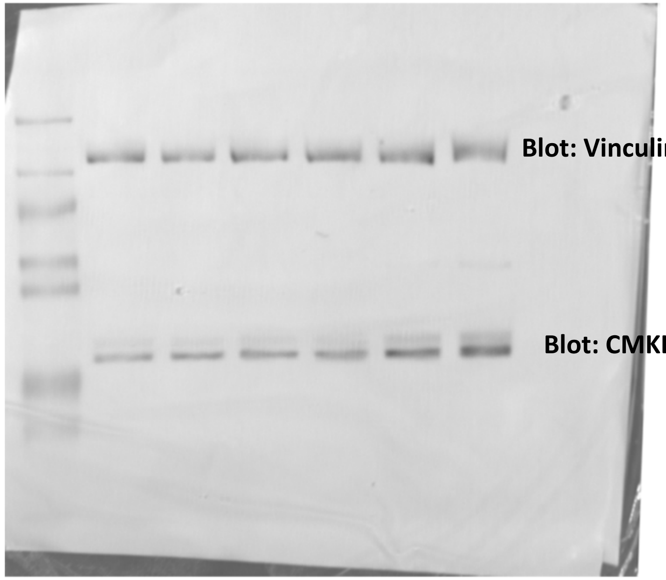

Figure 3B

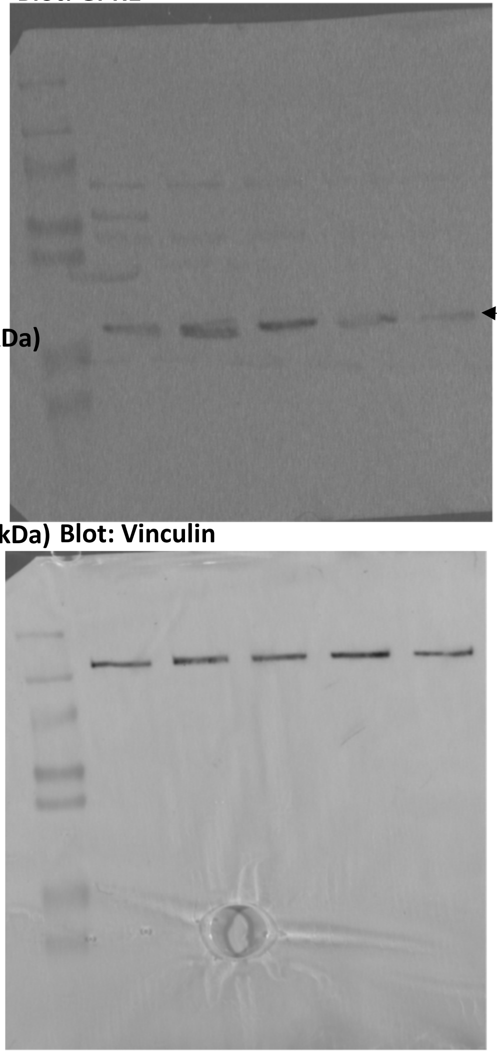

Figure 3C

This blot has been probed with another antibody (Akt) justifying a band around 56 kDa

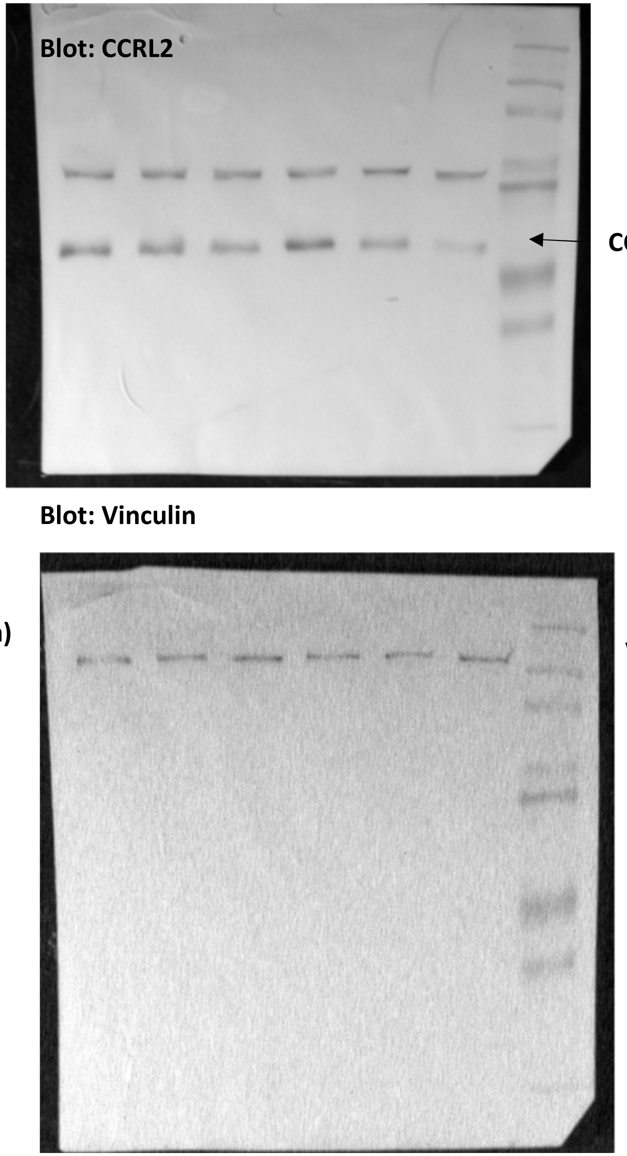

Figure 4A

Blot: Chemerin

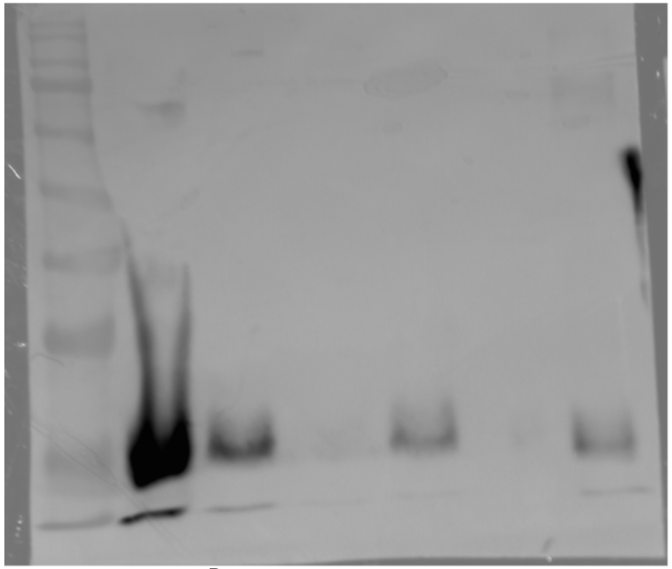

Ponceau

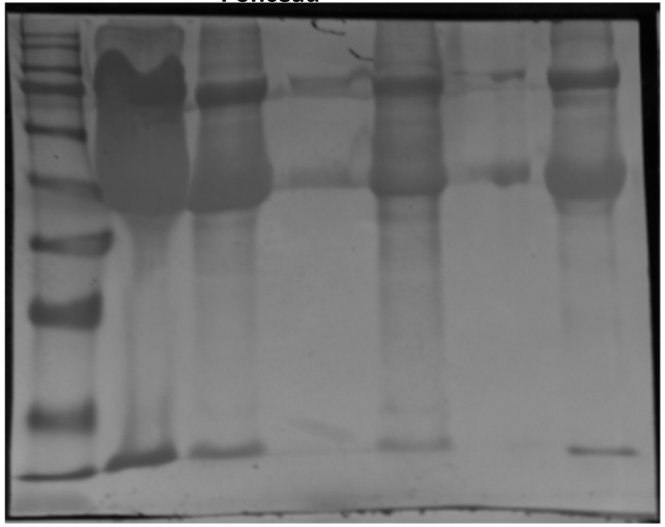

Blot: Chemerin (blot cropped)

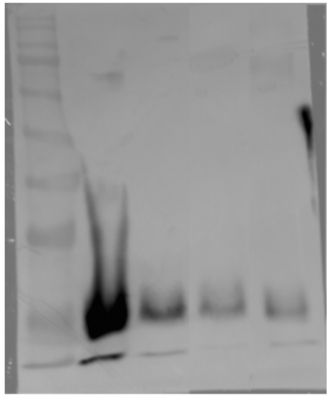

Ponceau cropped

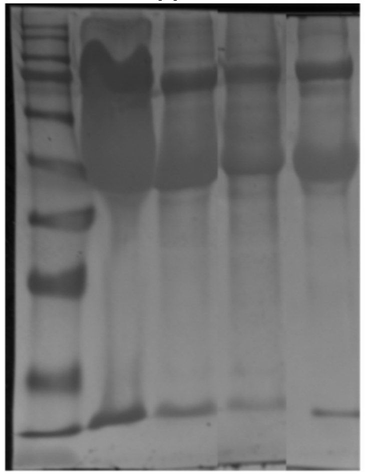

Figure 4C

Blot: Chemerin

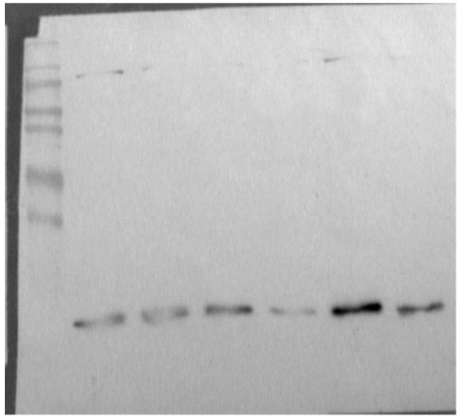

Blot: Vinculin

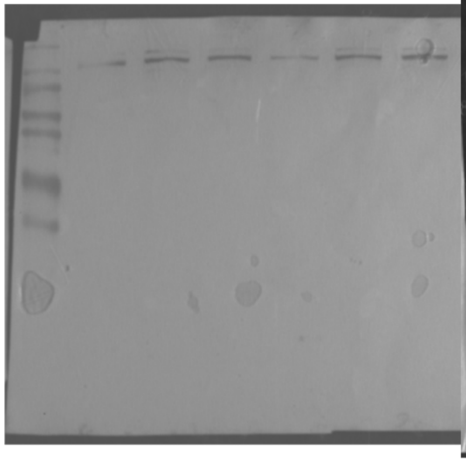

Figure 4D

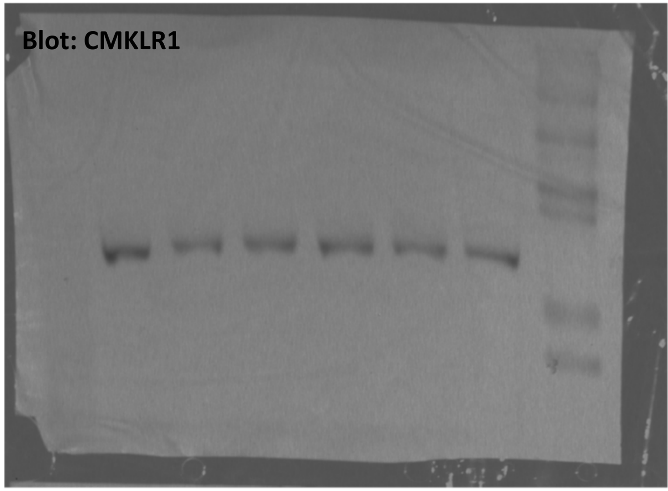

Blot: Vinculin

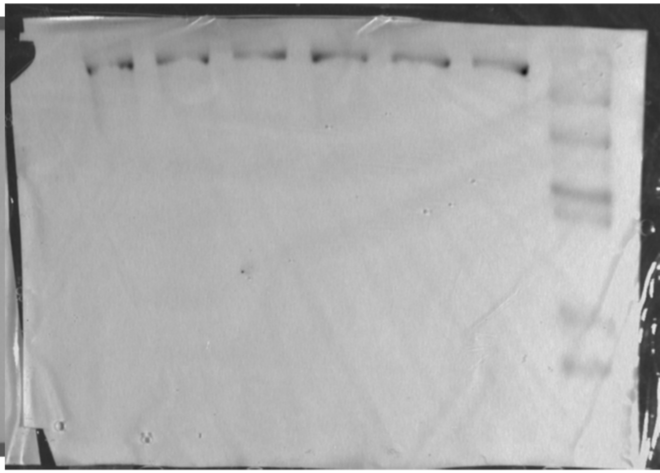

Figure 4E

Blot: GPR1

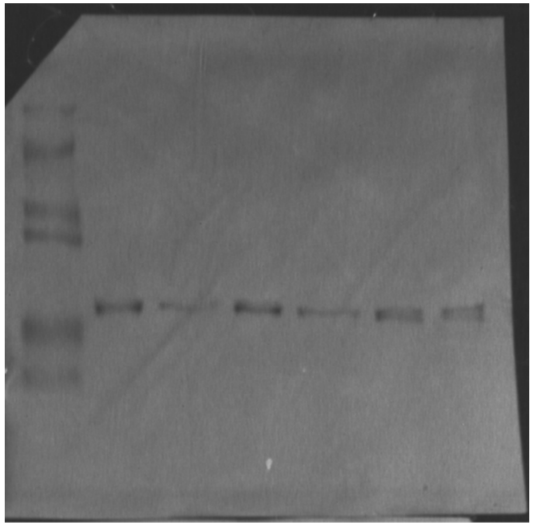

Blot: Vinculin

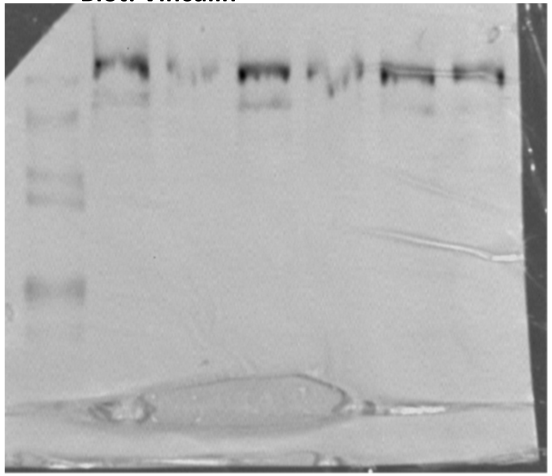

Figure 4F

Blot: CCRL2

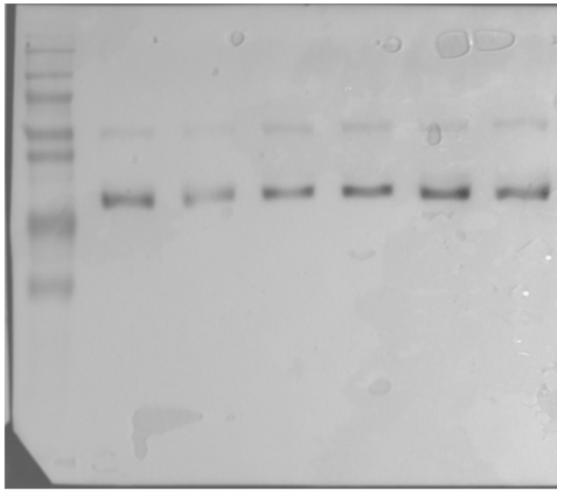

Blot: Vinculin

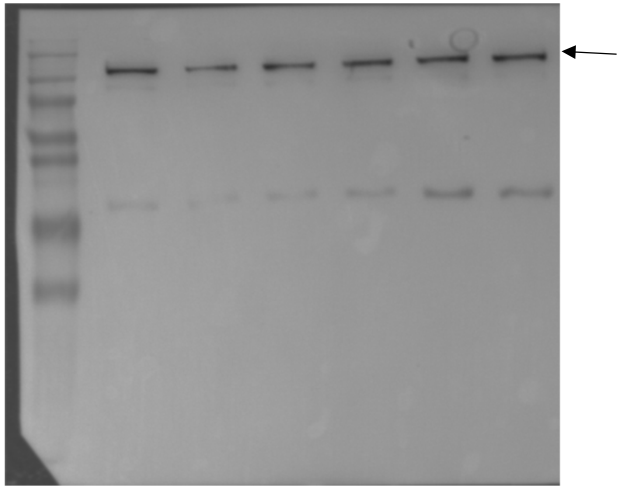

Vinculin

Figure 5A

Blot: Chemerin

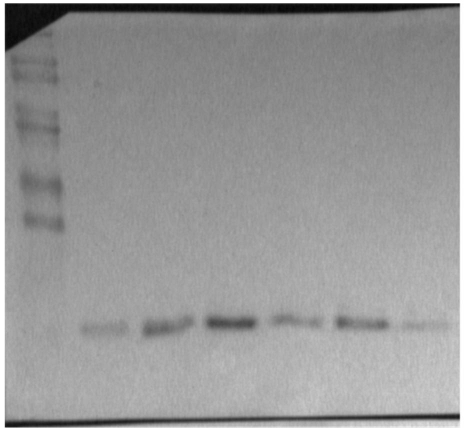

Blot: Vinculin

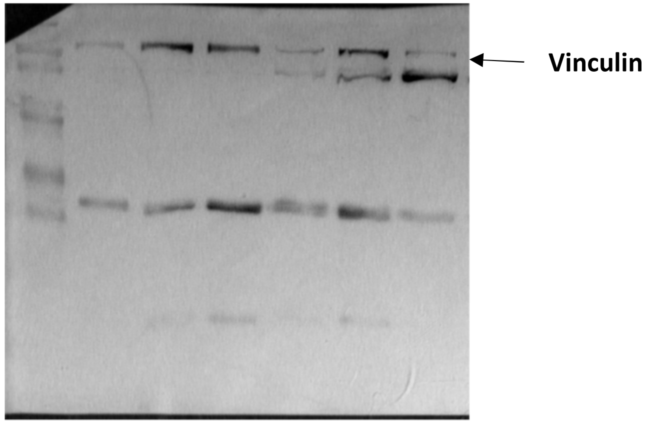

Figure 5B

Blot: CMKLR1

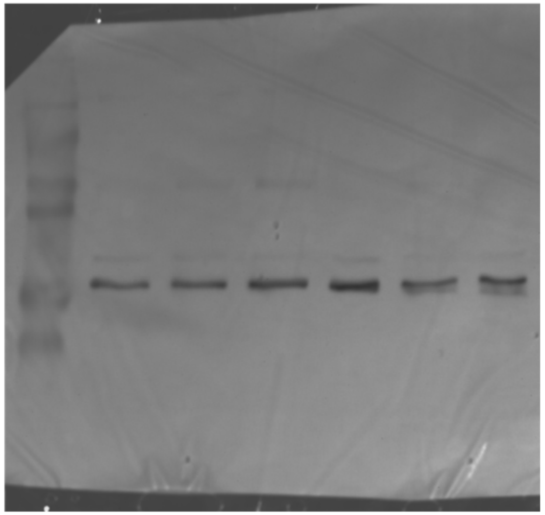

Blot: Vinculin

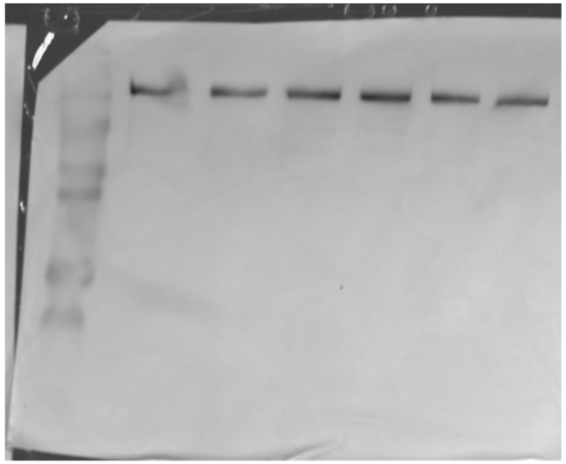

Figure 5C

Blot: GPR1

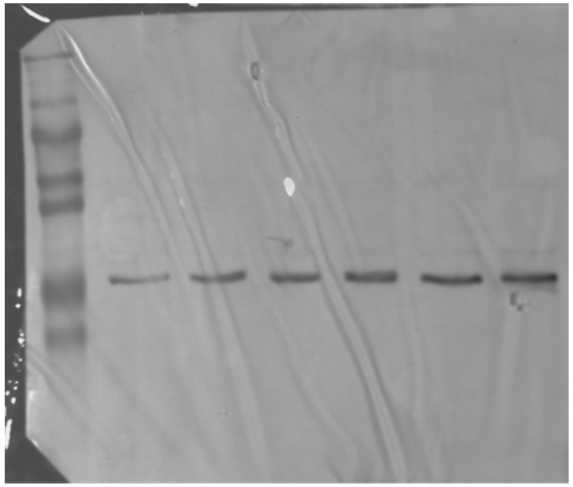

Blot: Vinculin

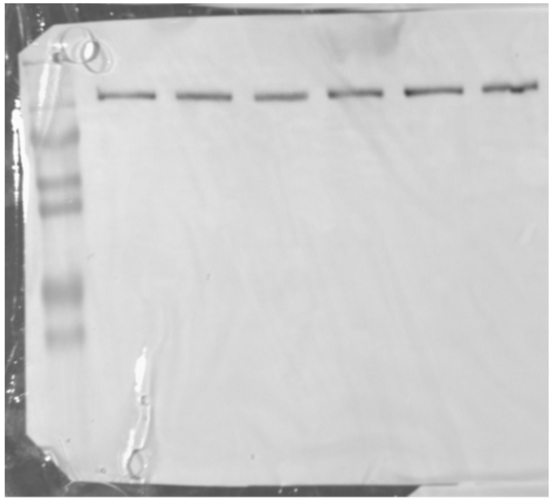

Figure 5D

Blot: CCRL2

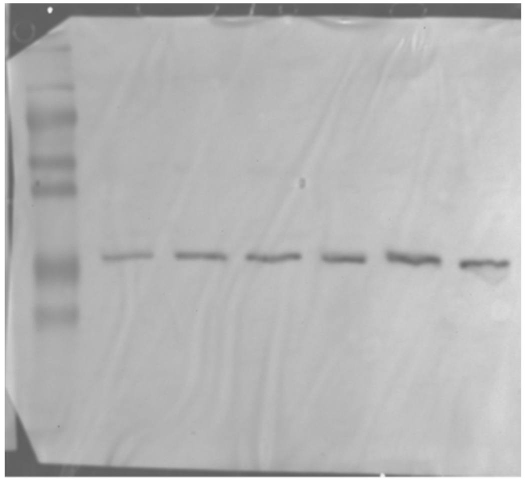

Blot: Vinculin

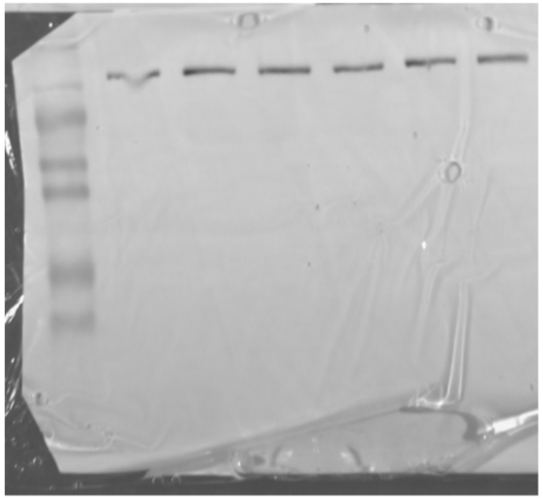

Figure 5E

Blot: Chemerin

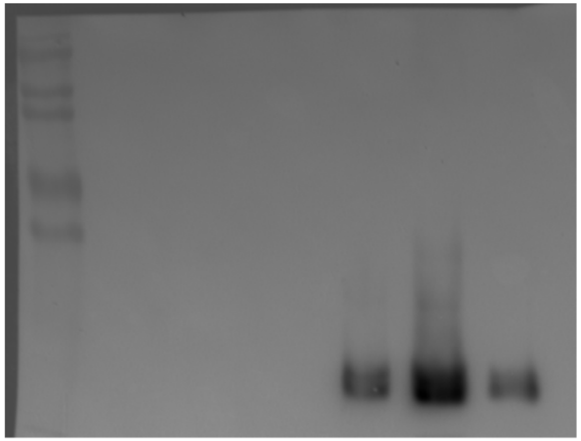

Blot: Ponceau

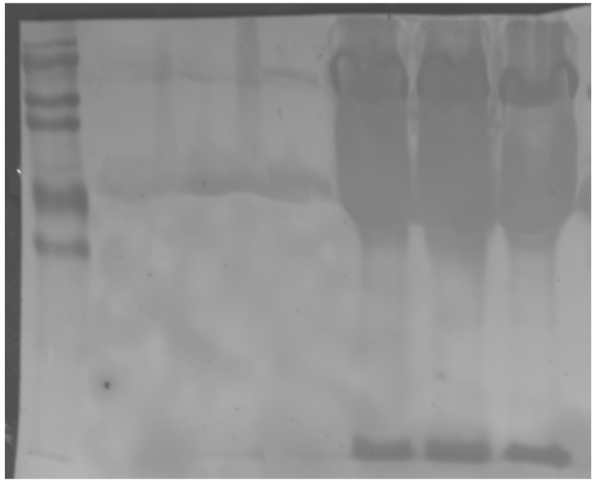

Supplement: Supplementary file 1 — Supplementary Figures. [file 41598_2022_12961_MOESM1_ESM.pdf]
